# Study on the alkylation of aromatic hydrocarbons and propylene

**DOI:** 10.3906/kim-2107-55

**Published:** 2021-12-06

**Authors:** Wanglai DONG, Wentao MA, Xinyao LI, Xingbang HU, Zheng ZHOU

**Affiliations:** School of Chemistry and Chemical Engineering, Nanjing University, Nanjing, China

**Keywords:** Aromatic hydrocarbon, propylene, alkylation, molecular sieve catalyst

## Abstract

In order to improve the efficiency of the alkylation reaction to aromatic hydrocarbons and propylene, different types of catalysts were screened, including ultra-stable Y molecular sieves (USY), solid phosphoric acid (SPA), ZSM type molecular sieve (HZSM), etc. The effects of reaction temperature, catalyst loading, and reaction time on the conversion rate of aromatic hydrocarbons and the selectivity of target products were investigated using the high-pressure reaction device. The catalysts were characterized by XRD, BET, SEM, FT-IR, NH_3_-TPD, and other methods. The experimental results show that the USY catalyst exhibits higher catalytic activity for alkylation. This catalyst can be used for the alkylation of different aromatic hydrocarbons. Good conversion and selectivity can be obtained. Futhermore, in a six-cycle experiment, the USY catalyst was reused without loss of efficiency.

## 1. Introduction

The alkylation reaction is a reaction process in which alkyl groups are introduced into the aromatic ring in the presence of a catalyst. As an important organic synthesis method, it is widely used in the synthesis of petrochemical products, drugs, perfumes, dyes and pesticides. In the process, propylene is used as an isopropylating agent for alkylation reaction, and the product cumene from the reaction with aromatic hydrocarbon compounds is used as an important industrial intermediate, which is widely used in petrochemicals, cosmetics, dyes, medicine, and many other large-scale synthesis of chemical industries [1–4].

At present, the isopropylation reaction to benzene and propylene has been widely used in the industrial production of phenol. However, there are few reports on the alkylation reaction of other aromatic hydrocarbons with propylene to produce phenolic, and the current reaction of benzene with propylene is usually carried out under the catalysis of homogeneous acid catalysts such as FeCl_3_, AlCl_3_, BF_3_, ZnCl_2_, HF, or H_2_SO_4_ [5–8]. Such catalysts have many disadvantages, such as difficulty in regeneration and post-treatment, inconvenient separation, corrosion, and poisoning [9]. In order to develop and find more efficient and environmentally friendly catalysts, the reusable solid catalyst with high activity has aroused great interest of researchers in recent years [10,11].

With the development of porous materials, molecular sieve catalysts have shown the advantages of good activity, no pollution, no corrosion, high shape selectivity, and good stability. As one of the key catalysts in many chemical processes of modern chemistry [12–17], it has gradually been widely used in alkylation reactions [18–20]

Therefore, the current research of many scholars is mainly focused on molecular sieve catalysts. In the alkylation reaction of benzene and propylene, many scientific research teams have done a lot of research [21,22]. The common synthesis process is the EniChem process, which uses a modified β zeolite catalyst. The process adopts a fixed-bed liquid phase method. The conversion rate of cumene is reached under the following conditions: n (benzene)/n (propylene) = 7.4, temperature = 150 °C, pressure = 3.0 MPa, and the yield reached = 99% [23].

In the toluene alkylation reaction, Selvaraj et al [24]. studied the catalytic toluene alkylation performance of Al-MCM-41 molecular sieves with different Si/Al (21-104). The experimental results showed that as the Si/Al value of Al-MCM-41 molecular sieve increase, the conversion rate of toluene gradually decreases. Paid et al [25]. studied the catalytic performance of three macroporous molecular sieves Hβ, HY, and HMCM-22 and found that the order of catalytic conversion of the catalyst is Hβ≈HY>HMCM-22, and the selectivity order of the target product is HMCM-22>HY> Hβ, comprehensive consideration of conversion rate and selectivity factor, namely HY, is the most suitable catalyst. Sridevi et al [26]. explored the catalytic activity and kinetics of the gas-phase alkylation reaction on Hβ zeolite molecular sieve. The reaction temperature is 210 °C, n(benzene)/n(isopropanol) = 8:1, and the mass space velocity is 4h^−1^; the conversion rate of isopropanol is 100%, and the selectivity of cumene is 93.55%, and it still has high catalytic activity after 400 h of operation. Reddy et al [27]. reported that the gas phase alkylation reaction of benzene and isopropanol can be carried out on Beta zeolite at a temperature of 150–270 °C, normal pressure, n(benzene)/n(isopropanol) = 8:1. On this basis, scholars successively began to develop better performance catalysts and explore better reaction conditions [28,29]. In another study on the alkylation of aromatic hydrocarbons, Kikuchi E et al [30]. used aluminosilicate and mordenite as catalysts to compare the liquid-phase alkylation reaction of naphthalene and propylene and found that, under the action of aluminosilicate, the selectivity of 2,6-diisopropylnaphthalene and 2,7-diisopropylnaphthalene is basically the same; the selectivity of 2,6-diisopropylnaphthalene under the action of mordenite is significantly greater than that of 2,7-diisopropyl naphthalene, and the selectivity gap increases with the increase of the Si/Al ratio of mordenite. Cresol[4, 31–36], but they did not mention the prospects of industrial application. Moreover, these studies have paid more attention to the isopropylation reaction of a single aromatic hydrocarbon, and few people have conducted systematic and summary studies on its process and catalyst universality.

**Scheme 1:**
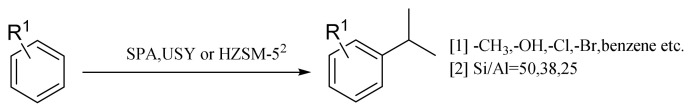
Friedel-Crafts process of aromatic hydrocarbons and propylene.

**Scheme 2:**
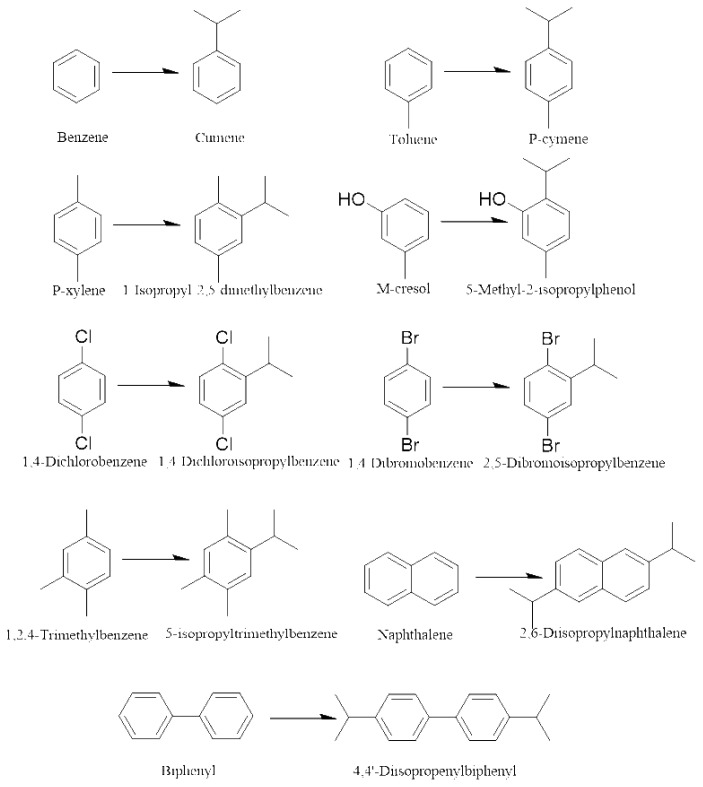
Main product of alkylation reaction of various aromatic hydrocarbons.

In this paper, the effects of reaction temperature and time on the catalytic activity, selectivity, and stability of various catalysts (SPA, USY, HZSM-5(Si/Al=25,38,50, the ratio of Si/Al is atomic ratio)) have been studied. The suitable reaction conditions were screened out through experiments, and the effect of molecular sieves with different Si/Al ratios on the alkylation reaction efficiency was explored. The effect of conversion rate on benzene, toluene, p-xylene, biphenyl, naphthalene, 1,4-dichlorobenzene, m-cresol, 1,2,4-trimethylbenzene, 1,4-dibromobenzene, and other compounds is summarized. The schematic diagram of the alkylation reaction and the main products are shown in Scheme 1–2.

## 2. Experimental section

### 2.1. Experiment reagent

Benzene (Analytical Reagent(AR)), toluene, p-xylene (AR), naphthalene (AR), Sinopharm Chemical Reagent Co., Ltd., propylene (≥99.9%), Nanjing Tianze Gas Co., Ltd., Biphenyl (AR) Shanghai Macleans Biochemical Technology Co., Ltd., 1,4-dichlorobenzene (AR), m-cresol (AR), 1,2,4-trimethylbenzene (AR), 1,4-dibromobenzene (AR), Shanghai Minrell Chemical Technology Limited company. HZSM-5, Beijing Huawei Ruike Chemical Co., Ltd. USY, Zibo Runxin Chemical Technology Co., Ltd. SPA, Liaoning Haitai Technology Co., Ltd.

### 2.2. Experimental procedure

The alkylation reaction was performed in a 50mL high-pressure reactor. Reaction condition: 0.1 mol of aromatic hydrocarbon compounds, 5wt%–25wt% of catalyst powder, and 0.8MPa of propylene. The magnetic rotor is used, and the magneton rotation speed is set to 600rpm. The reaction time is 24 h (with 6 h as the reaction interval, 4 times of propylene is charged in to ensure the excess reaction of propylene). The reaction temperature is set to 160–240°C, and the temperature is kept constant during the whole reaction. After the reaction, the composition of raw materials and products was analyzed using a SHIMADZU GC-2014C gas chromatograph, which was equipped with a WondaCap-5 capillary column (30 m × 0.32 mm × 0.25 μm) and a flame ionization detector. The product was determined by MS.

## 3. Results and discussion

### 3.1. Kinetics analysis

**Figure 1:**
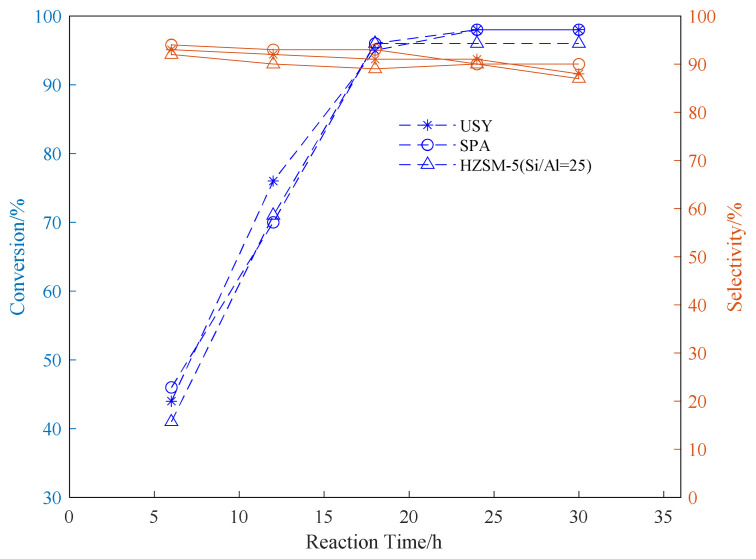
Conversion and selectivity of benzene alkylation reaction at different reaction time.

**Figure 2:**
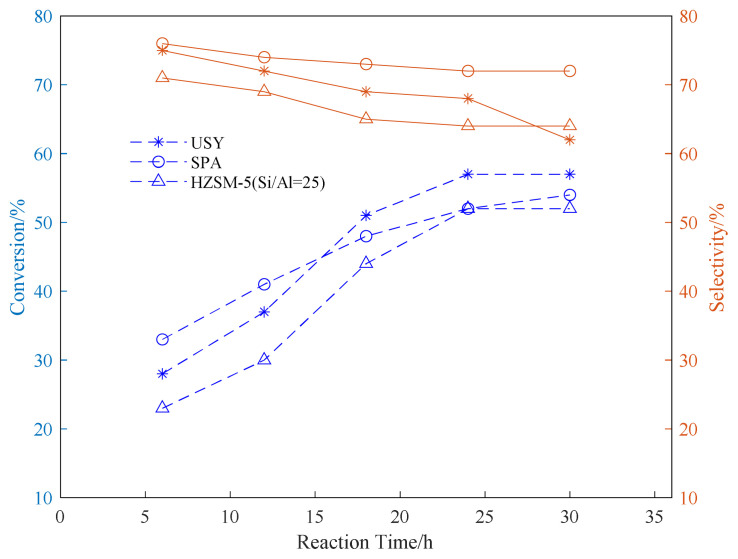
Conversion and selectivity of toluene alkylation reaction at different reaction time.

**Figure 3:**
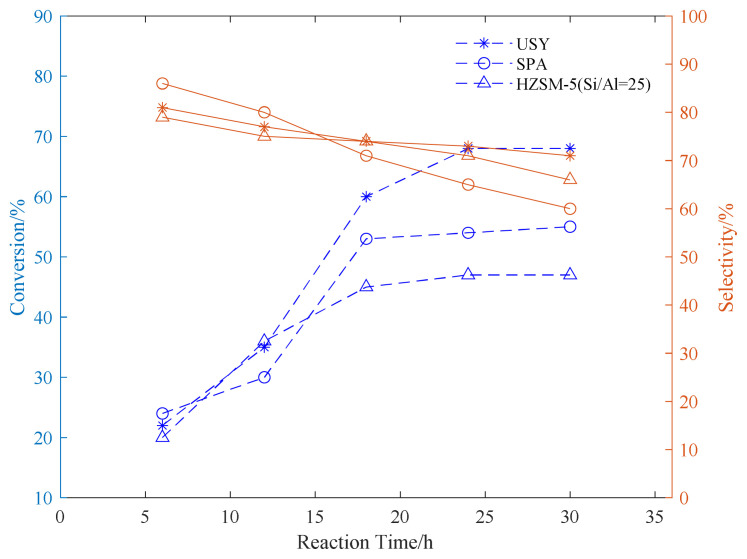
Conversion and selectivity of p-xylene alkylation reaction at different reaction time.

Using SPA, USY, and HZSM-5 as catalysts, three common aromatic hydrocarbons, benzene, toluene, and p-xylene, were selected to explore the appropriate conditions for the reaction. In the exploration of the reaction time, the reaction temperature was fixed at 180 °C. The propylene ventilation pressure is 0.8 MPa, and the catalyst loading is 20wt%. The effect of reaction time on the conversion rate of alkylation raw materials and product selectivity is explored. The results are shown in Figures 1–3.

It can be seen from the data in the Figures 1–3 that as the reaction time increases, the conversion rate of raw aromatic hydrocarbons under the same pressure and reaction temperature will increase. The conversion rate of the reactants remains basically unchanged after 24 h of reaction. USY gave higher conversion compared to SPA and HZSM-5. In terms of the conversion rate of the target product, it can be seen from the figure that the selectivity of aromatics decreases with the increase of reaction time, we infer that as the reaction time increases, the alkylation products produced by the reaction begin to accumulate and occupy the pores of the catalyst, thereby affecting the selectivity of the target product[37].

To investigate the influence of reaction temperature on the reaction, the ventilation pressure of propylene is fixed to 0.8 MPa, the catalyst loading is 20wt%, the reaction time is set to 24 h, and the investigation range of reaction temperature is 160–240 °C.

**Figure 4:**
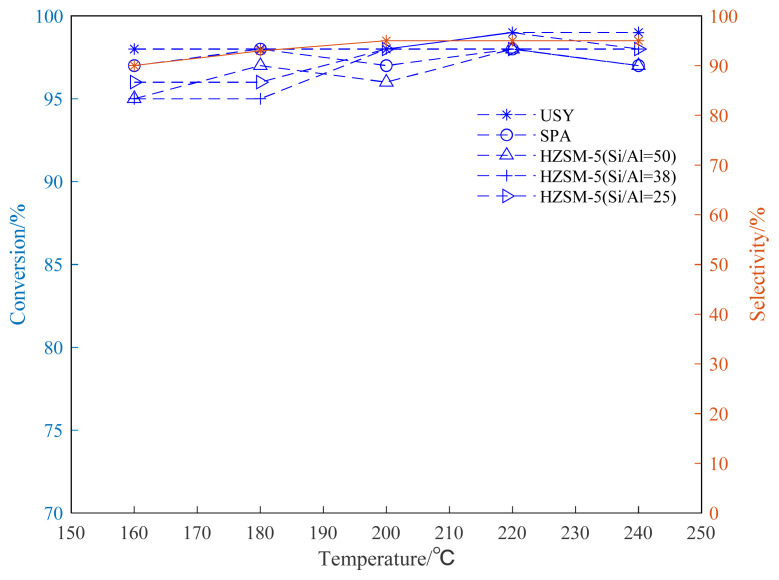
Conversion and selectivity of benzene alkylation reaction at different reaction temperatures.

**Figure 5:**
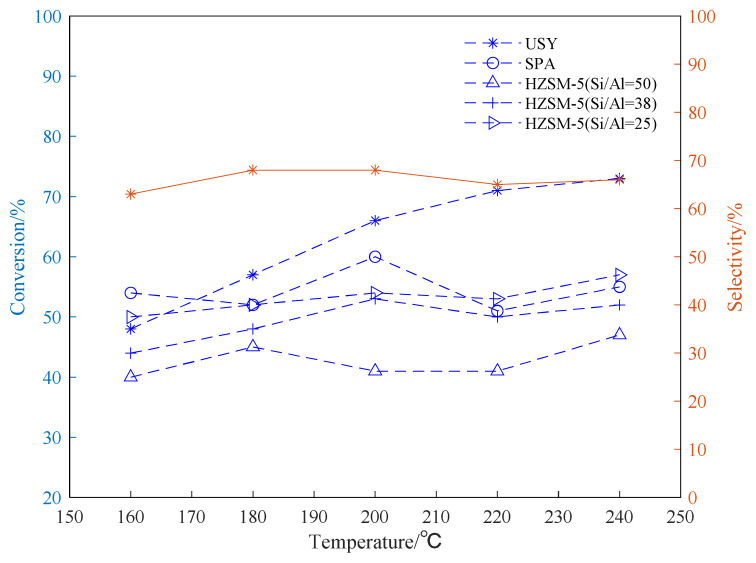
Conversion and selectivity of toluene alkylation reaction at different reaction temperatures.

**Figure 6:**
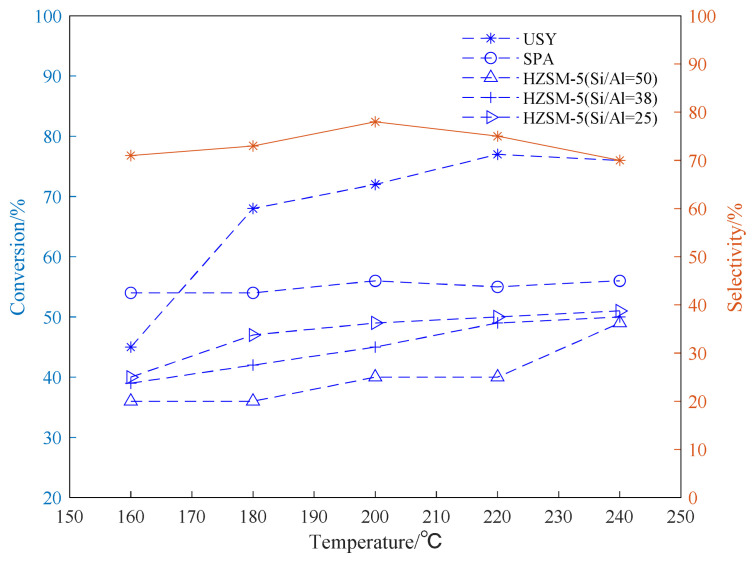
Conversion and selectivity of p-xylene alkylation reaction at different reaction temperatures.

The alkylation reaction of propylene and aromatic hydrocarbon compounds is an endothermic reaction [38, 39], and high temperature is conducive to the progress of the reaction. However, as the temperature increases, we infer that the reaction between aromatic hydrocarbons and propylene becomes complicated, and side reactions such as isomerization, overalkylation, and polymerization intensify. Products and side reactions affect the activity of the catalyst, which, in turn, leads to a decrease in the selectivity of target products [40–42]. It can be seen from Figures 4–6, the selectivity of the target product of the alkylation reaction of benzene, toluene, and p-xylene under the USY catalysis shows a trend of first increasing and then decreasing with the increase of the reaction temperature.

Figures 4–6 show the effect of temperature on the conversion rate of benzene, toluene, and p-xylene and the selectivity of the target product in the presence of different types of catalysts. The graphical results show that the order of catalytic effect is USY>SPA>HZSM-5(25)>HZSM-5(38)>HZSM-5(50). Using USY, the highest conversion rate of benzene, toluene, and p-xylene can reach 99, 73 and 77%, respectively. Under this conversion rate, the selectivities of the target products cumene, p-cymene, and 2,5-dimethyl cumene are 95, 66, and 75%, respectively.

Based on the results of three different HZSM-5 (Si/Al=25,38,50) molecular sieve catalysts, it can be seen that smaller Si/Al (Si/Al=25) can bring higher conversion rate of raw materials.

The aromatic hydrocarbon alkylation reaction mainly goes through the following processes: (1) the diffusion of reactant molecules from the gas phase to the outer surface of the catalyst, (2) the diffusion of reactant molecules in the catalyst pores, the activity of reactant molecules in the catalyst pores, (3) chemical reaction of reactant molecules on the active center in the catalyst pore, (4) desorption of product molecules from the active center in the catalyst pore, (5) the diffusion of product molecules away from the outer surface of the catalyst to the main body of the gas phase. Hence, the pore size of the catalyst directly affects the reaction. It was found that the catalytic effect of the USY molecular sieve with a larger pore size is significantly better than that of the HZSM-5 molecular sieve with a smaller pore size. It can be inferred that when HZSM-5 catalyzes the reaction, the reactants or products diffusion were limited by its pores, so that the catalyzed reaction can only rely on the limited acidic center on the outer surface, thus affecting its catalytic effect. The USY site is better than HZSM-5. The site is easier to reach and therefore exhibits higher catalytic activity [43, 44].

It can be seen from Figure 4 that, in the alkylation reaction between benzene and propylene, almost 100% conversion rate of benzene can be obtained using USY. The reaction of benzene and propylene to cumene is mature in industry and has been widely used in industry [23, 45, 46]. It is worth noting that the catalytic conversion rate of the USY molecular sieve is slightly better than other catalysts, and it can be seen from the figure that under USY catalysis, the target monosubstituted cumene product selectivity is above 90%.

From the results shown in Figure 5, it can be seen that catalytic reactivity of the USY in the toluene alkylation reaction is significantly better than other catalysts, which is consistent with the results of the benzene alkylation reaction shown in Figure 4. The toluene conversion rate can reach up to 73%. In addition, in the presence of HZSM-5 with different Si/Al, lower Si/Al (Si/Al=25) can lead to higher toluene conversion rate. The highest selectivity of the target mono-substituted product p-cymene using USY is about 68%. The difference from the benzene alkylation reaction is that the toluene alkylation reaction exhibits a decrease in selectivity when the temperature is too high (220–240 °C). The possible reason is that when the temperature is too high, the reaction tends to generate m-cymene and o-cymene, which, in turn, reduces the selectivity of the target product, p-cymene.

From the results shown in Figure 6, it can be seen that the catalytic reactivity of the USY catalyst in the alkylation reaction of p-xylene and propylene is significantly better than those of other catalysts. The change trend of the conversion rate is basically the same as that of the alkylation reaction of benzene and toluene. Consistent, the conversion rate can reach up to 77%. In addition, under the catalysis of HZSM-5 with different Si/Al, lower Si/Al (25) brings better effect of catalyzing the isopropylation reaction. Being consistent with the results of the benzene alkylation and toluene alkylation reaction, the USY catalyst exhibits a better catalytic effect. Using USY, the target monosubstituted 2,5-dimethylcumene has a maximum selectivity of about 78%. The difference between the alkylation reaction is that when the temperature of the para-xylene alkylation reaction is too high (220–240 °C), the selectivity of the target product decreases. This may be due to the fact that the catalyst catalyzes the 2,5-dimethylcumene when the temperature is too high. The di-substitution reaction of cymene is intensified, which reduces the selectivity of the target product.

In addition, when the Si/Al ratio of HZSM-5 catalyst is 25, the conversion rate of toluene alkylation reaction is 57%, and when the Si/Al ratio of HZSM-5 catalyst is 50, the conversion rate of toluene alkylation reaction is 47%. The experimental results are basically consistent with the results of p-xylene alkylation.

**Figure 7:**
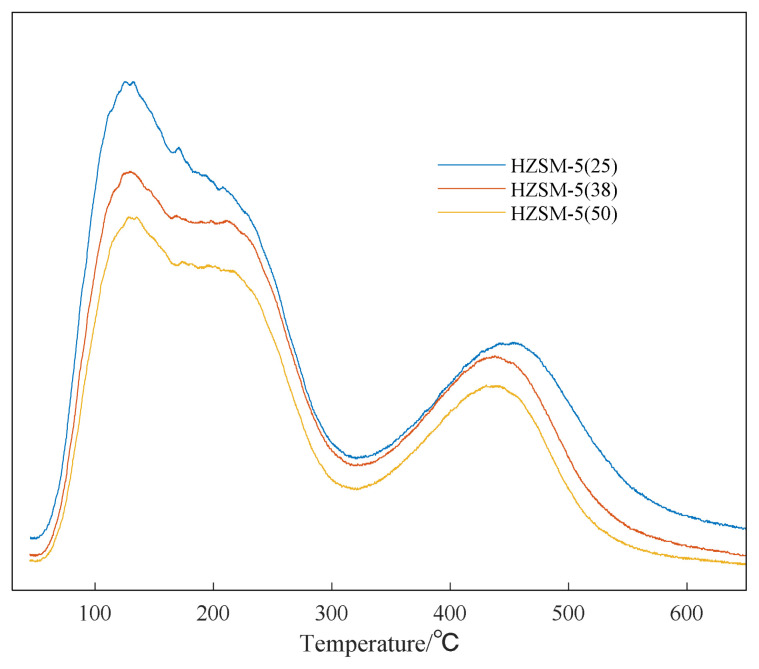
NH_3_-TPD characterization result of HZSM-5 catalyst.

In order to explore the catalytic performance of HZSM-5 with different silicon to aluminum ratios, NH_3_-TPD was used to characterize the surface acid properties of HZSM-5. As shown in Figure 7, HZSM-5 has two obvious NH_3_ desorption peaks. The NH_3_ desorption temperature is lower than 300 °C as the weak acid center, and the NH_3_ desorption temperature is 450–550°C as the strong acid center [47]. The number of weak acid and strong acid sites of HZSM-5 with different silicon-to-aluminum ratios can be obtained by integrating the NH_3_-TPD spectrum [48]. It can be seen from the Figure 7 that, as the ratio of silicon to aluminum increases, the number of acidic sites, weak acid and strong acid strength of HZSM-5 are reduced.

Combined with the FT-IR characterization of Figure S5, the adsorption peaks between 3500–400cm^−1^ are in line with the typical skeleton characteristic peaks of HZSM-5 molecular sieve [49–52]. It can be seen that the HZSM-5 stretching vibration peaks with different silicon-to-aluminum ratios at 3414cm^−1^ have a significant difference, the peak at 3414 cm^−1^ corresponds to the Al-OH framework (Brønsted acid sites) [53]. It can be seen from Figure S6 that all HZSM-5 have unique diffraction peaks of the MFI crystal structure in the low angle 7.5°–8.8° and high angle 22°–25°. The position of the diffraction characteristic peak is 7.91°, 8.88°, 23.04°, 24.05°, 24.58°[54].

From the above analysis, it can be seen that as the ratio of silicon to aluminum increases, the weaker the acid strength of HZSM-5, the worse the catalytic effect [55].

In summary, in the above three aromatic hydrocarbons reactions, comprehensive analysis of aromatic hydrocarbon conversion and selectivity shows that reaction performed at 220 °C gives better result, so the subsequent reaction experiment temperature is set to 220 °C. In addition, in the investigation of the reaction time and reaction temperature, we found that the USY catalyst is significantly better than other catalysts, so we will directly use the USY catalyst for subsequent investigations.

### 3.2. Optimization of USY catalyst loading

On the basis of determining the reaction time and temperature, we explored the effect of the amount of catalyst on the conversion rate of raw materials and product selectivity. Part of the characterization data of the ultra-stable Y-type molecular sieve in this paper is shown in supporting information. The specific surface area of the USY ultra-stable molecular sieve is determined by N_2_ adsorption at −196°C, and the specific surface area is 350.0m^2^/g. The rest refer to support information for the characterization content.

**Figure 8:**
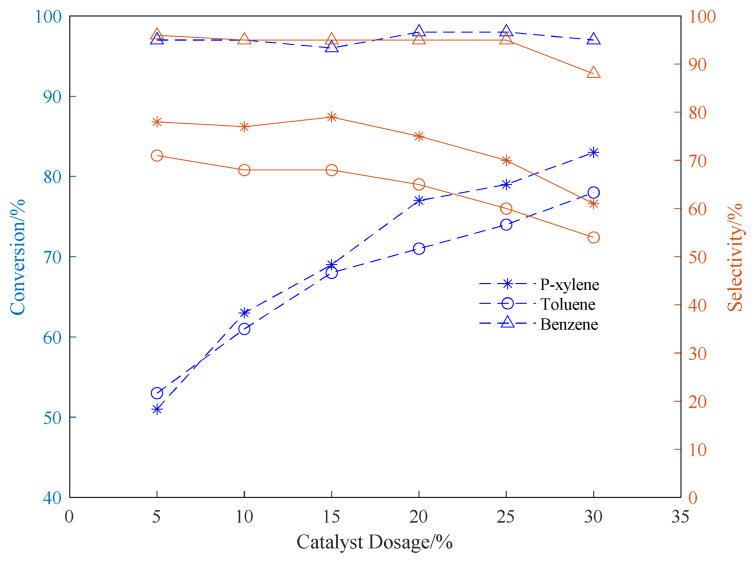
Conversion and selectivity of aromatic hydrocarbon alkylation reaction under different catalyst dosage. (The ventilation pressure of the fixed propylene was 0.8MPa, the reaction time was set to 24h, and the reaction temperature was set to 220°C.)

Figure 8 shows that as the amount of catalyst increases, the conversion rate of aromatic hydrocarbons also increases at the same time. This is because the alkylation reaction of aromatic hydrocarbons with propylene is mainly electrophilic substitution on the benzene ring, which belongs to the carbocation ion mechanism. Propylene is activated into isopropyl carbocation under the action of protic acid (H^+^), and then the isopropyl carbocation ion can react with aromatic hydrocarbons to form isopropyl aromatic hydrocarbons.^14^ The carbocation reaction mechanism supports that the more the amount of catalyst used, the more acidic active sites the catalyst can be provided, and the higher the concentration of carbocation produced per unit time, the higher the conversion rate of reaction raw materials. However, the increase in the amount of catalyst will lead to a decrease in the selectivity of the reaction product and more by-products. When the amount of catalyst exceeds 20wt%, it can be seen that the selectivity of the target product has a significant decline. When the catalyst loading is 20wt%, the selectivities of the reaction products of benzene, toluene, and p-xylene are 95, 65 and 75%, respectively. When the catalyst loading is 30wt%, the selectivities of the reaction products of benzene, toluene, and p-xylene are 88, 54 and 61%, respectively.

In addition, when the amount of catalyst is small, we infer that the reaction raw materials are easy to block the pores of the zeolite, thereby affecting the reaction conversion rate. Therefore, as the amount of the catalyst increases, the effect of the blockage of the pores on the deactivation of the zeolite decreases, and the reaction conversion rate increases accordingly. Considering the change trend of selectivity and conversion rate, the usage amount of the catalyst is 20wt% for follow-up experiments.

### 3.4 Research on the recyclable performance of USY catalyst

**Figure 9:**
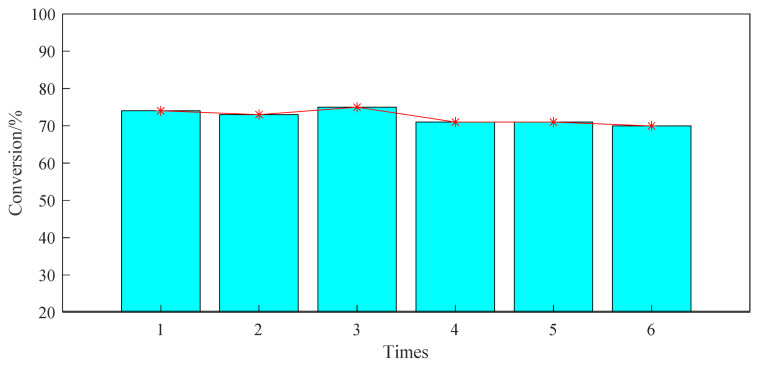
Catalytic performance of USY used repeatedly in alkylation reactions.

Based on the investigation of the above reaction conditions, the ventilation pressure of the fixed propylene is 0.8 MPa, the reaction time is set to 24h, and 20wt% of the USY of the raw aromatic hydrocarbon is added at 220 °C. The reusable performance of the USY molecular sieve was studied in a high-pressure reactor. After the experiment was completed according to the experimental procedure in the ‘Experimental procedure’, the product was centrifuged and filtered. The obtained filter cake was washed with acetonitrile, and the washing was repeated 5 times. Then, after drying the obtained filter cake catalyst, the experiment was repeated according to the above reaction steps. It can be seen from the experimental results in Figure 9 that the USY catalyst is recycled for 6 times, and its catalytic reactivity is almost unchanged.

**Figure 10:**
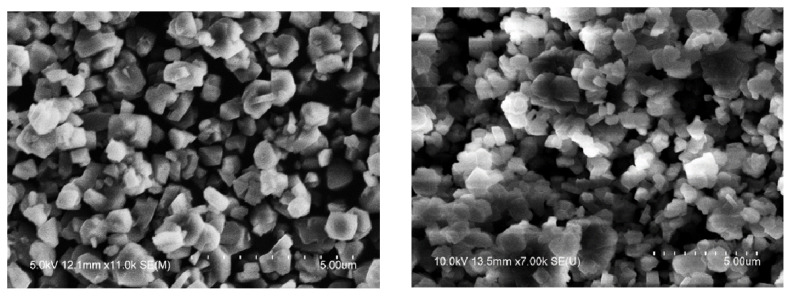
SEM of unused (left) and 6-time used (right) USY.

It can be seen from the SEM image in Figure 10 that the molecular sieve was almost nonwearing after 6 reactions in the high-pressure reaction device. The USY molecular sieve particles showed irregular octahedral shape, regular appearance, smooth surface, and small particles. After repeated reactions 6 times, the conversion rate of raw materials and their SEM pictures have not changed significantly.

**Figure 11:**
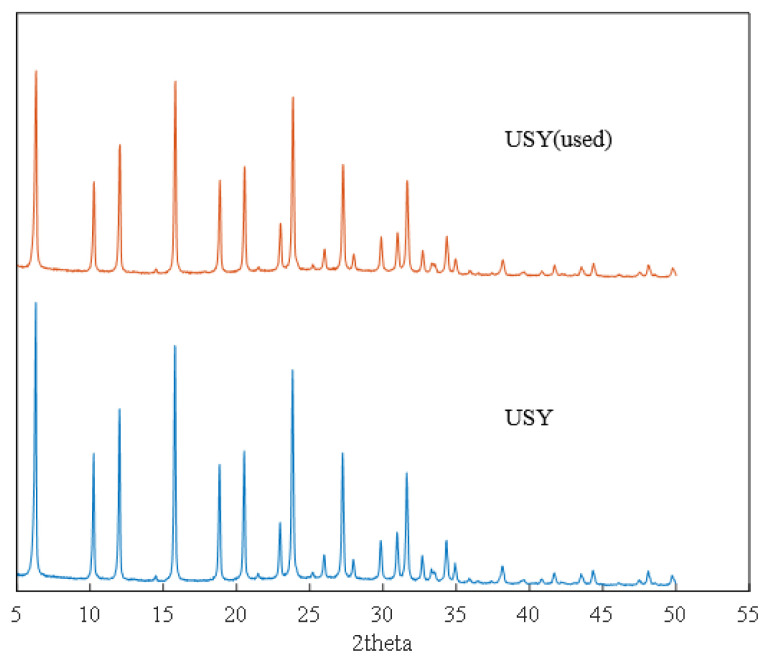
XRD characterization result of unused and 6-time used USY.

**Figure 12:**
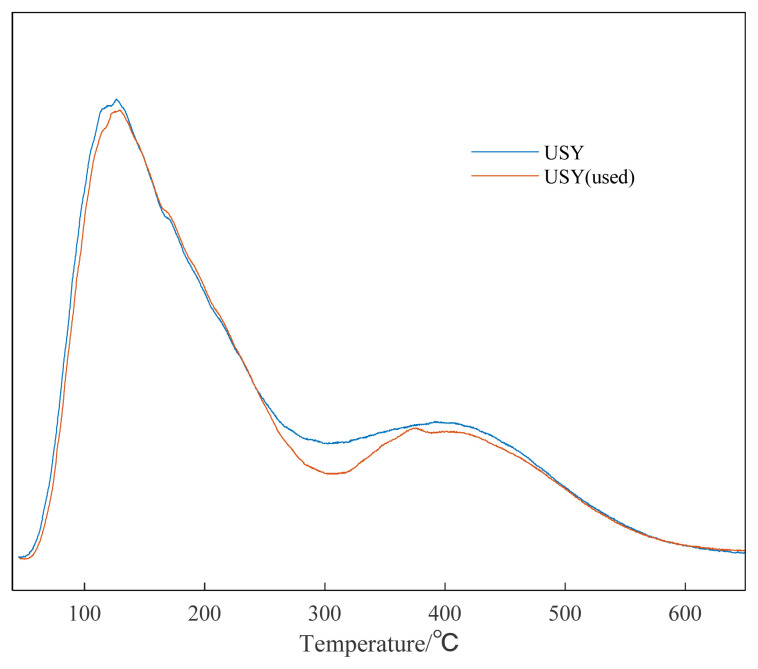
NH_3_-TPD characterization result of unused and 6-time used USY.

In addition, it can be seen from Figure S1 and Figure S2 that the adsorption and desorption curve and pore size distribution of the recycled catalyst hardly change, and the specific surface area has changed from 350.0 m^2^/g to 315.8 m^2^/g. This may be due to the blockage of some catalyst gaps after the reaction. The fresh USY catalyst and the used USY were also characterized by XRD. It can be seen from the XRD pattern that USY molecular sieve is dominated by FAU molecular sieve crystal phase, with obvious crystal structure and basic framework structure of molecular sieve. After six cycles of use, it can be seen from Figure 11, the alkylation reaction basically did not destroy the original crystal structure of USY, with sharp diffraction peaks and high crystallinity. NH_3_-TPD was used to characterize the surface acid properties of the USY catalyst. It can be seen from figure 12 that the total amount of surface acid remained basically unchanged after USY was used six times.

Therefore, in summary, the USY catalyst has certain practical application significance and potential industrial application prospects in the aromatic hydrocarbon alkylation reaction.

### 3.3. Research on the universality of USY catalyst

**Figure 13:**
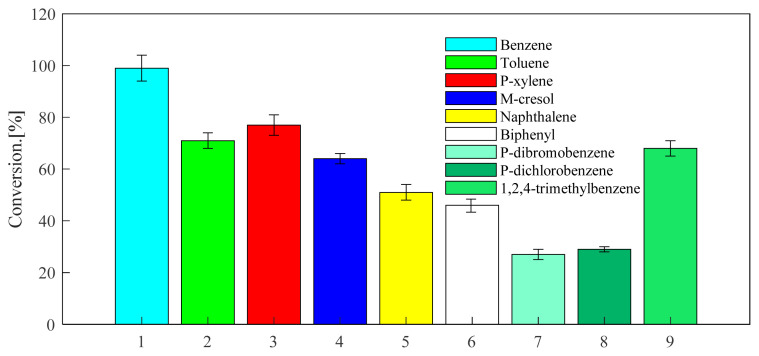
Conversion of different aromatics at 220 °C under USY catalysis. (The ventilation pressure of the fixed propylene was 0.8MPa, the reaction time was set to 24 h, the reaction temperature was set to 220 °C, and the catalyst loading is 20wt%).

**Figure 14:**
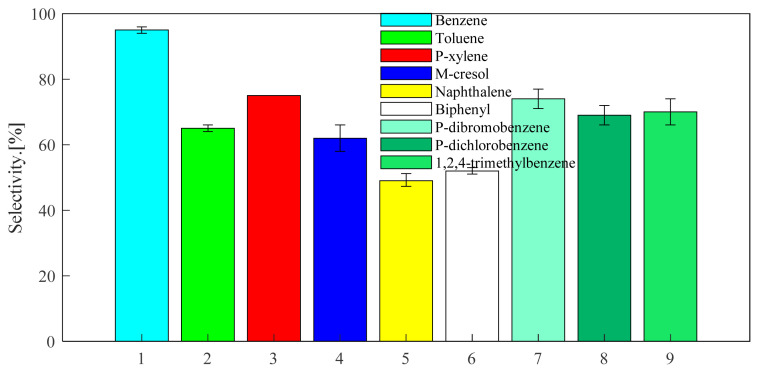
Selectivity of different aromatics at 220°C under USY catalysis. (The ventilation pressure of the fixed propylene was 0.8MPa, the reaction time was set to 24 h, the reaction temperature was set to 220 °C, and the catalyst loading is 20wt%).

Through the above exploration of benzene, toluene and p-xylene, the selected catalysts and optimized reaction conditions are applied to the alkylation reaction of other aromatic hydrocarbons with propylene. The screened aromatic hydrocarbons are currently in wide demand in the industry. The general applicability of the substrate was explored. The aromatic hydrocarbon substrates selected were naphthalene, biphenyl, m-cresol, 1,2,4-trimethylbenzene, 1,4-dichlorobenzene, 1,4-dibromobenzene. As can be seen from Figure 13, the reactivity of USY for different aromatic hydrocarbons is good. The USY catalyst catalyzes the alkylation conversion rates of naphthalene, biphenyl, m-cresol, 1,2,4-trimethylbenzene, 1,4-dichlorobenzene, 1,4-dibromobenzene and propylene to 51, 46, 64, 68, 29, and 27%, respectively. Considering Scheme 2 and Figure 14, it can be seen that the selectivity of the target product corresponding to each aromatic hydrocarbon alkylation is 49, 52, 62, 70, 69, and 74%, respectively. It has certain industrial application prospects.

## 4. Conclusion

The USY molecular sieve shows good catalytic performance for a variety of aromatic hydrocarbon compounds, and it can be recycled, which has certain industrial application prospects.

## Characterization results

### 1. The BET characterization result of USY catalyst

The specific surface area of the USY ultra-stable molecular sieve is determined by the BET theory. The specific surface area of the USY ultra-stable molecular sieve is determined by N_2_ adsorption at −196°C. The unused USY specific surface area is 350.0 m^2^/g, the used USY specific surface area is 315.8 m^2^/g. It can be seen from the figure that the N_2_ adsorption-desorption isotherm of USY molecular sieve is type I isothermal. The line is a typical microporous structure. The USY molecular sieve exhibits a wide range of hysteresis loops between the relative pressure of 0.1 to 0.8, indicating that its structure has a relatively developed mesoporous system.

The specific surface area, pore volume, and pore size distribution of the catalyst were measured on the ASAP 2020 type physical adsorption instrument from Micromeritics

Figure 1 BET characterization result of unused USY catalyst.

Figure 2 BET characterization result of used USY catalyst.

### 2. The FT-IR characterization result of USY catalyst

It can be seen from the figure that 455cm^−1^ corresponds to the T-O (T=Si/Al) bending vibration shrinkage peak; 575cm^−1^ is the characteristic peak of the USY molecular sieve Al-O tetrahedral double six-membered ring structure. 1170cm^−1^ is used for Si-O tetrahedral asymmetric stretching vibration peak, near 1397cm^−1^ corresponds to the Al-OH single bond stretching vibration absorption peak; the absorption peak corresponding to 1631cm^−1^ is the Si-OH single bond stretching vibration peak, and 3442cm^−1^ is the stretching of the interlayer water molecule vibration; 3617cm^−1^ is the acidic bridging hydroxyl group in the supercage.

FT-IR analyses were performed on Gangdong FT-IR-650 spectrometer with a highly sensitive DLATGS detector.

Figure 3 FT-IR characterization result of USY catalyst.

Figure 4 FT-IR characterization result of used USY catalyst.

### 3. The XRD and FT-IR characterization result of HZSM-5 catalyst

The phase structure of the catalyst was determined on the D8 Advance X-ray diffractometer of the German company Bruker.

Figure 5 FT-IR characterization result of used HZSM-5 catalyst.

Figure 6 XRD characterization result of HZSM-5 catalyst.

### 4. The NH3-TPD characterization result of USY catalyst and HZSM-5 catalyst

The temperature programmed desorption (TPD) patterns with ammonia on the samples were recorded on Microtrac BETCAT-A.

Figure 7 NH_3_-TPD characterization result of USY catalyst and HZSM-5 catalyst

### 4. The GC-MS trace of target product

The structures of the product and byproducts were further identified by using GC-MS (type: GC-TOF).

Figure 8 The GC-MS trace of cumene.

Figure 9 The GC-MS trace of P-cymene.

Figure 10 The GC-MS trace of 1-Isopropyl-2,5-dimethylbenzene.

Figure 11 The GC-MS trace of 5-Methyl-2-isopropylphenol.

Figure 12 The GC-MS trace of 5-Isopropyltrimethylbenzene.

Figure 13 The GC-MS trace of 4,4′-Diisopropenylbiphenyl.

Figure 14 The GC-MS trace of 2,6-Diisopropylnaphthalene.

Figure 15 The GC-MS trace of 2,5-Dibromoisopropylbenzene.

Figure 16 The GC-MS trace of 1,4-Dichloroisopropylbenzene.

Figure 17 The main products and by-products of the alkylation reaction of benzene, toluene, p-xylene, and propylene.
