# Supplementary figures and images for: Study on the alkylation of aromatic hydrocarbons and propylene
Source: Turk J Chem. 2021 Dec 6;46(2):446–58. doi: 10.3906/kim-2107-55 (PMC10734739; doi:10.3906/kim-2107-55)

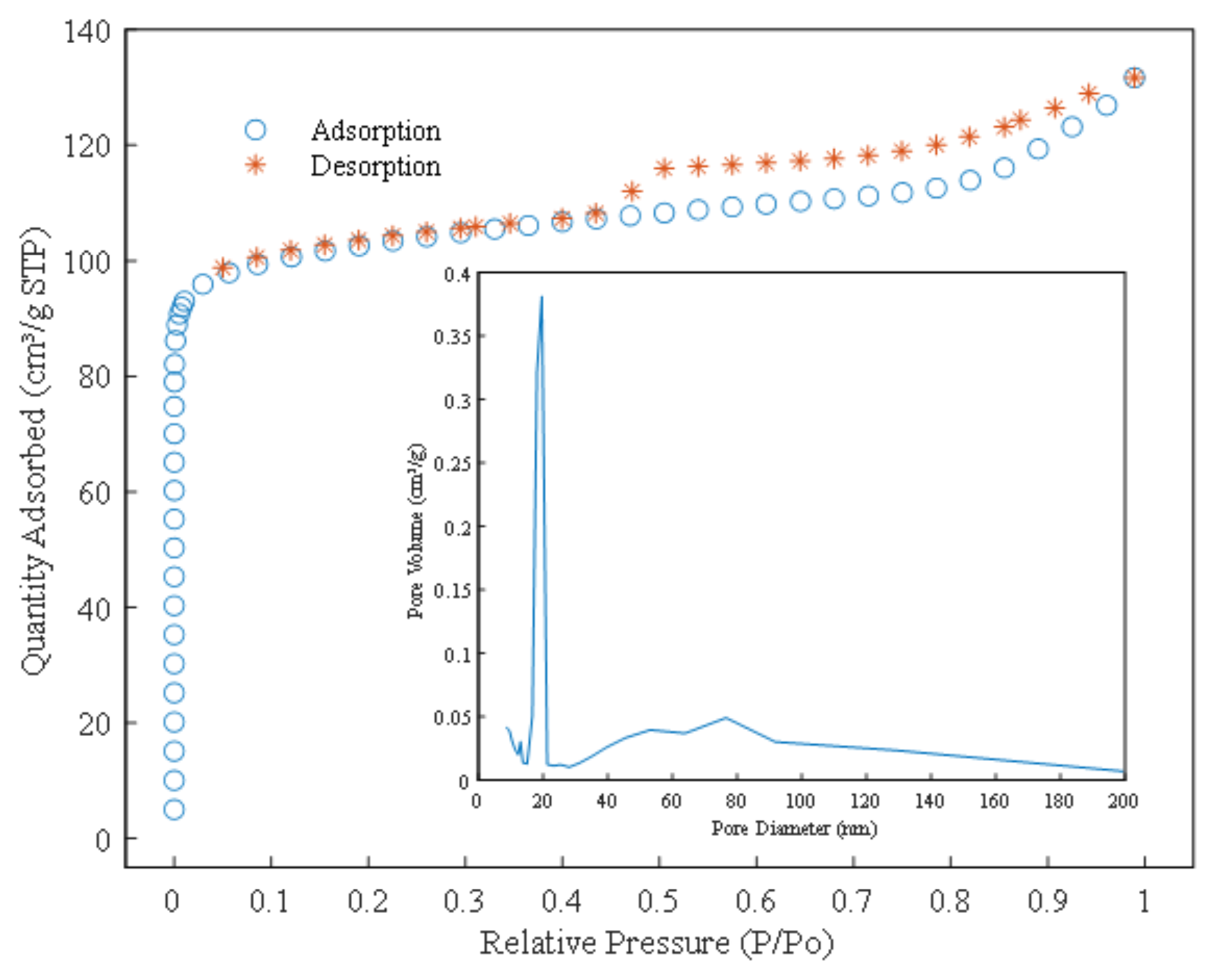

Supplement: Figure 1 — BET characterization result of unused USY catalyst. [file turkjchem-46-2-446s1.tif]

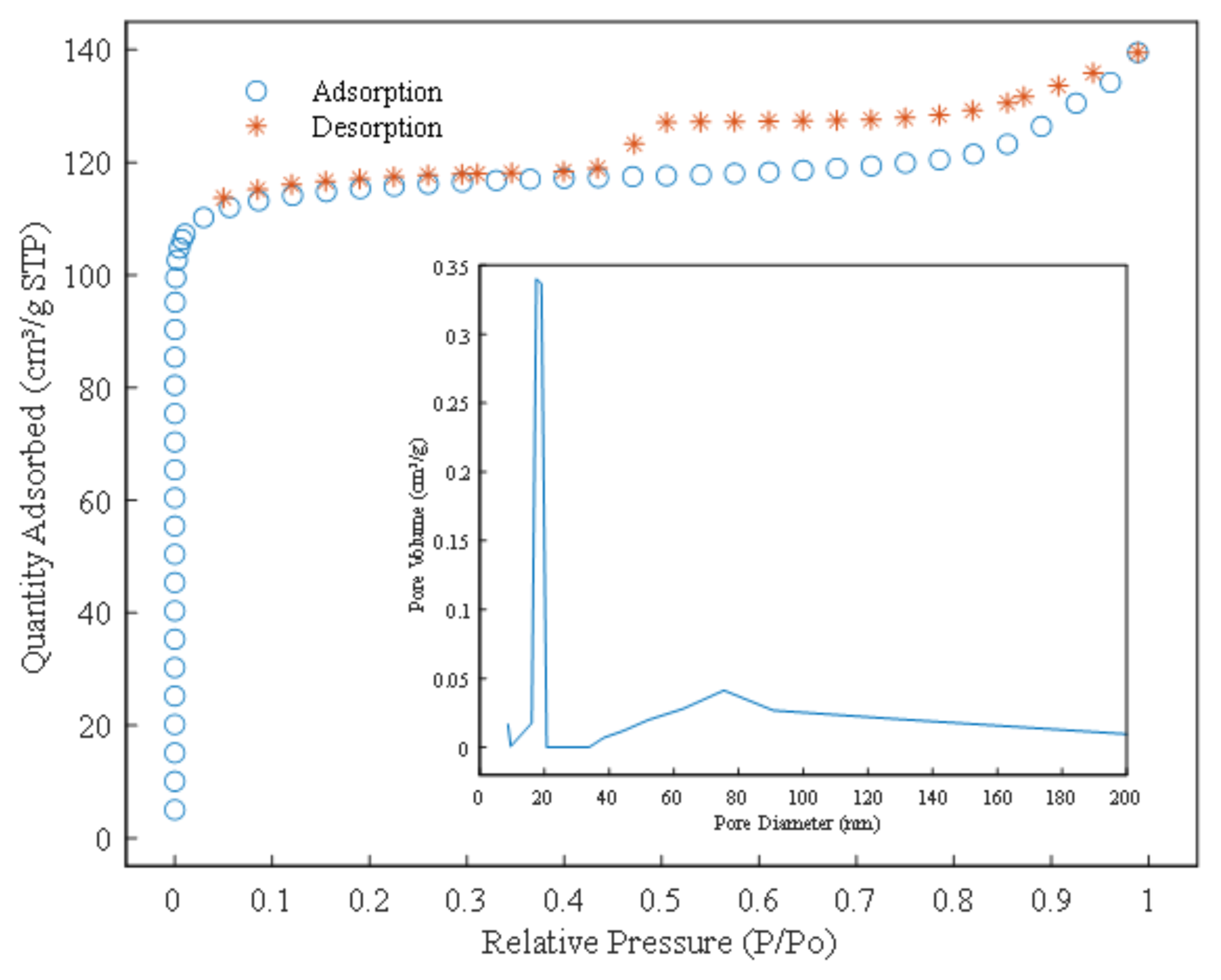

Supplement: Figure 2 — BET characterization result of used USY catalyst. [file turkjchem-46-2-446s2.tif]

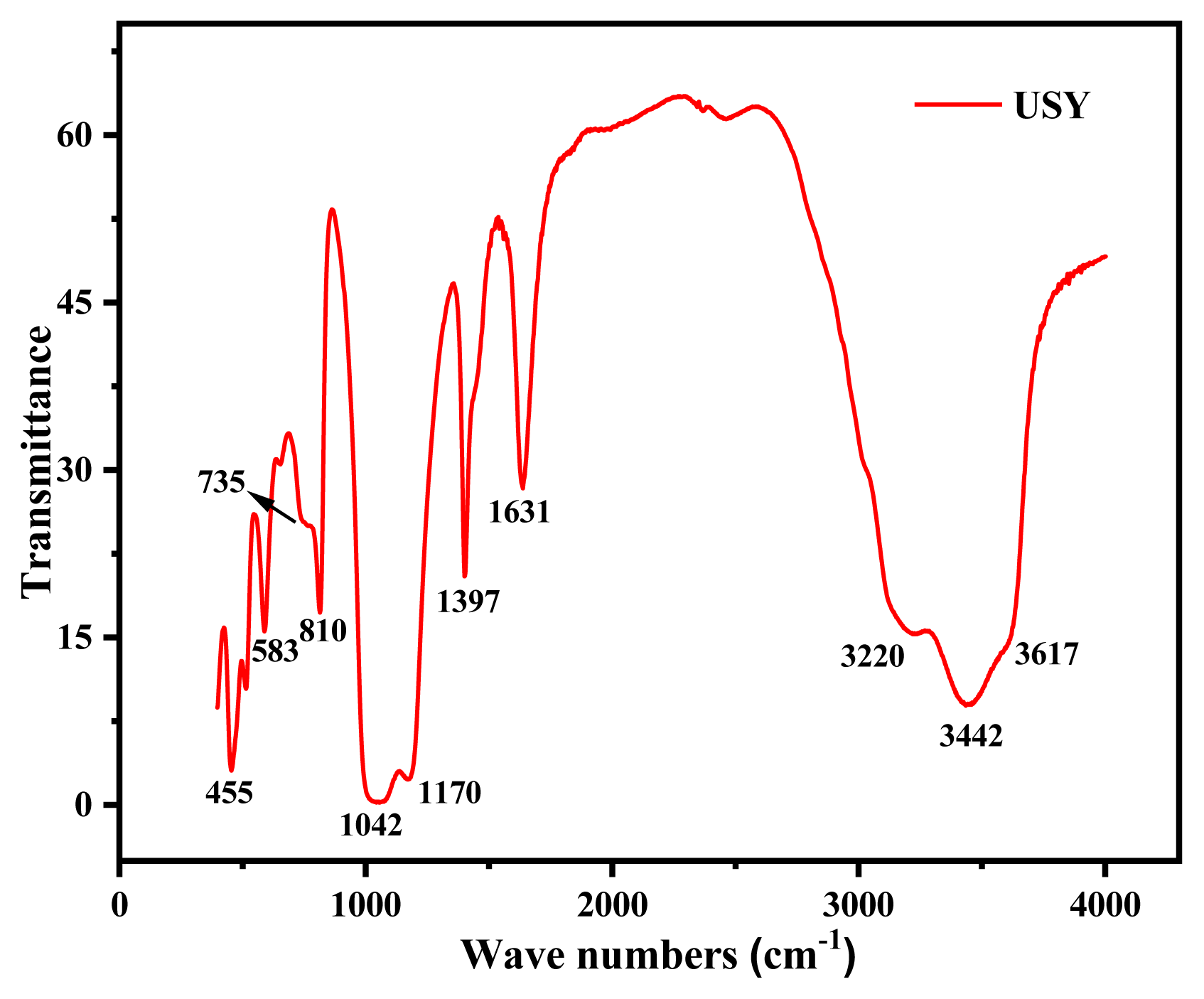

Supplement: Figure 3 — FT-IR characterization result of USY catalyst. [file turkjchem-46-2-446s3.tif]

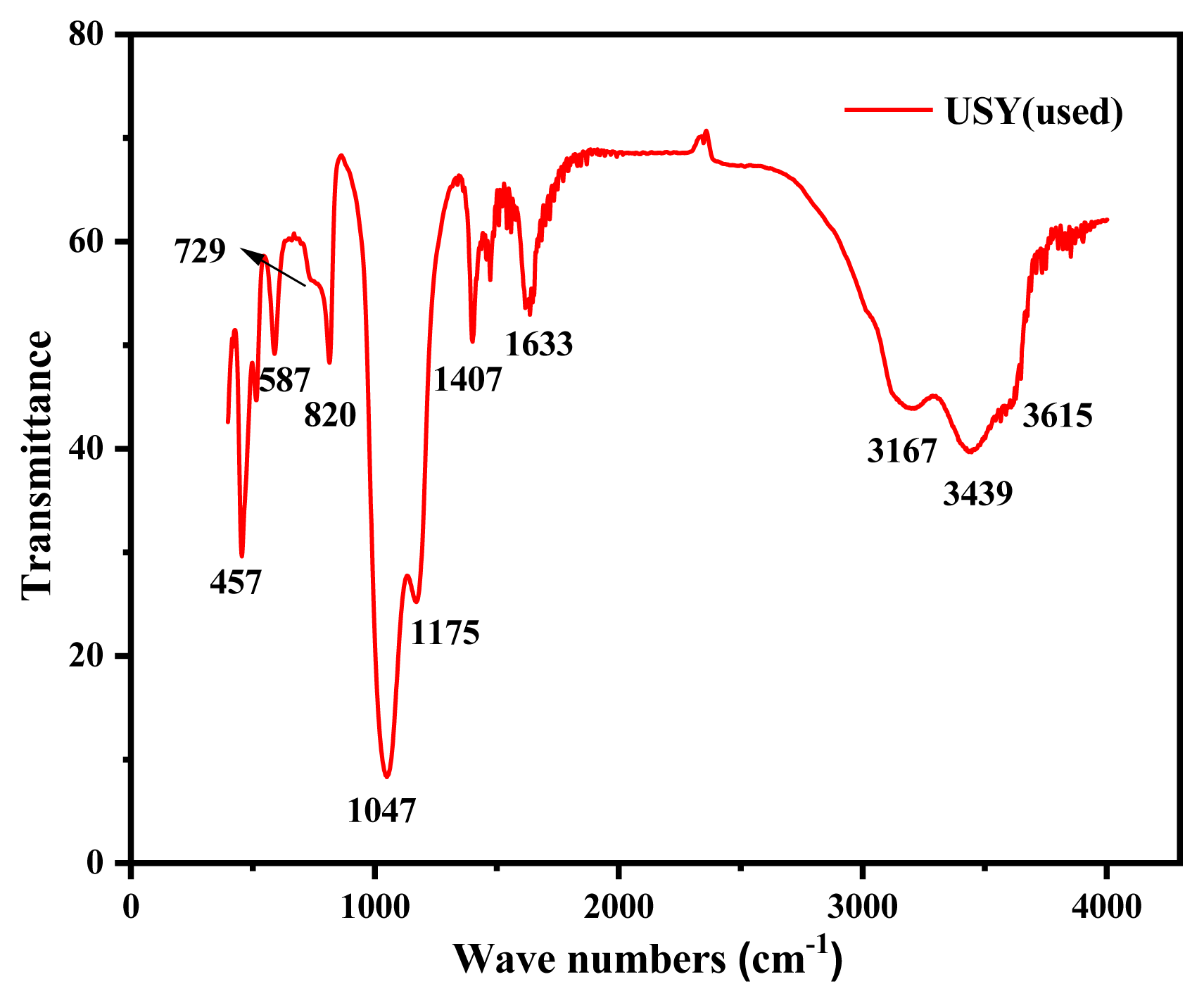

Supplement: Figure 4 — FT-IR characterization result of used USY catalyst. [file turkjchem-46-2-446s4.tif]

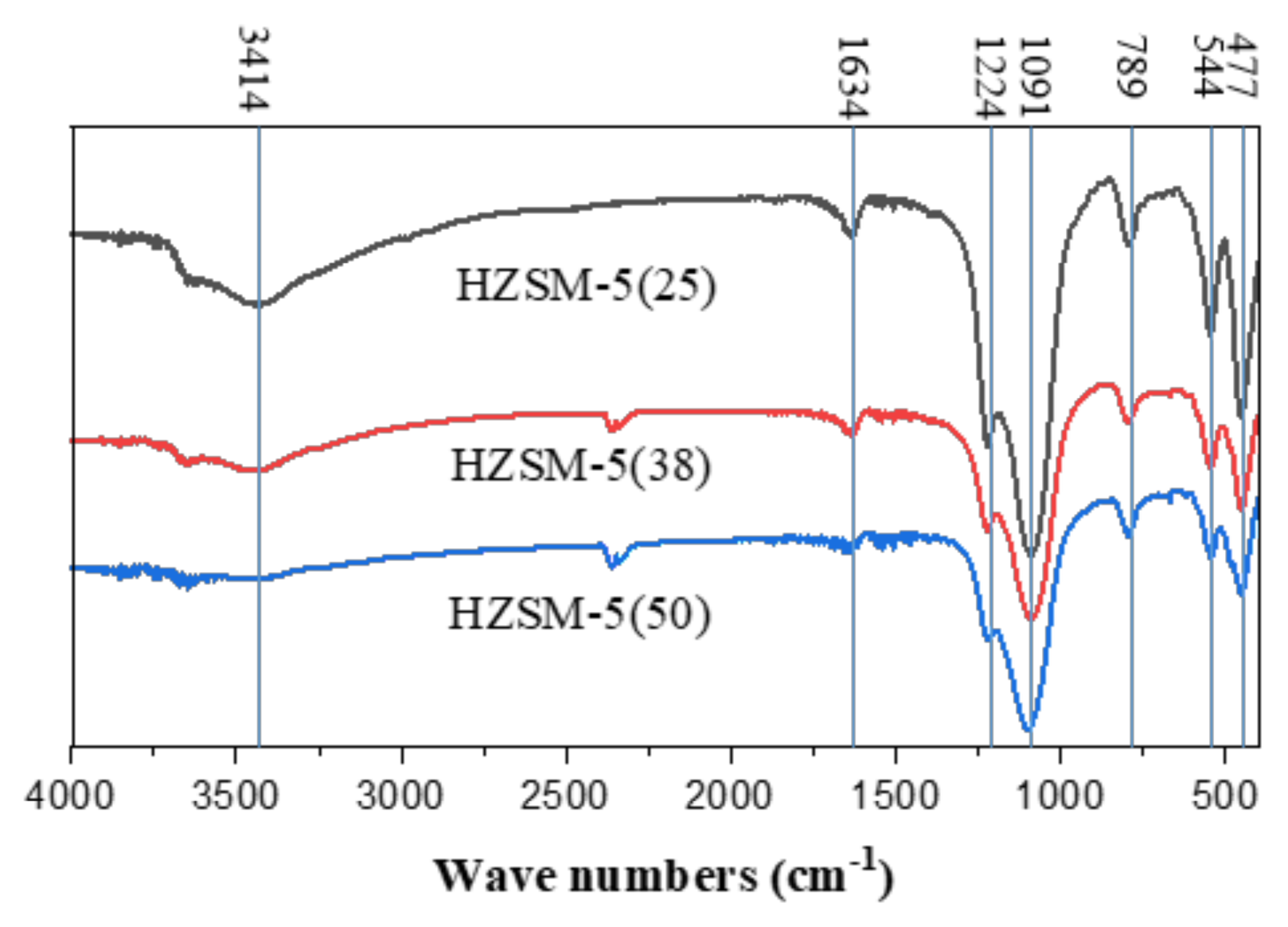

Supplement: Figure 5 — FT-IR characterization result of used HZSM-5 catalyst. [file turkjchem-46-2-446s5.tif]

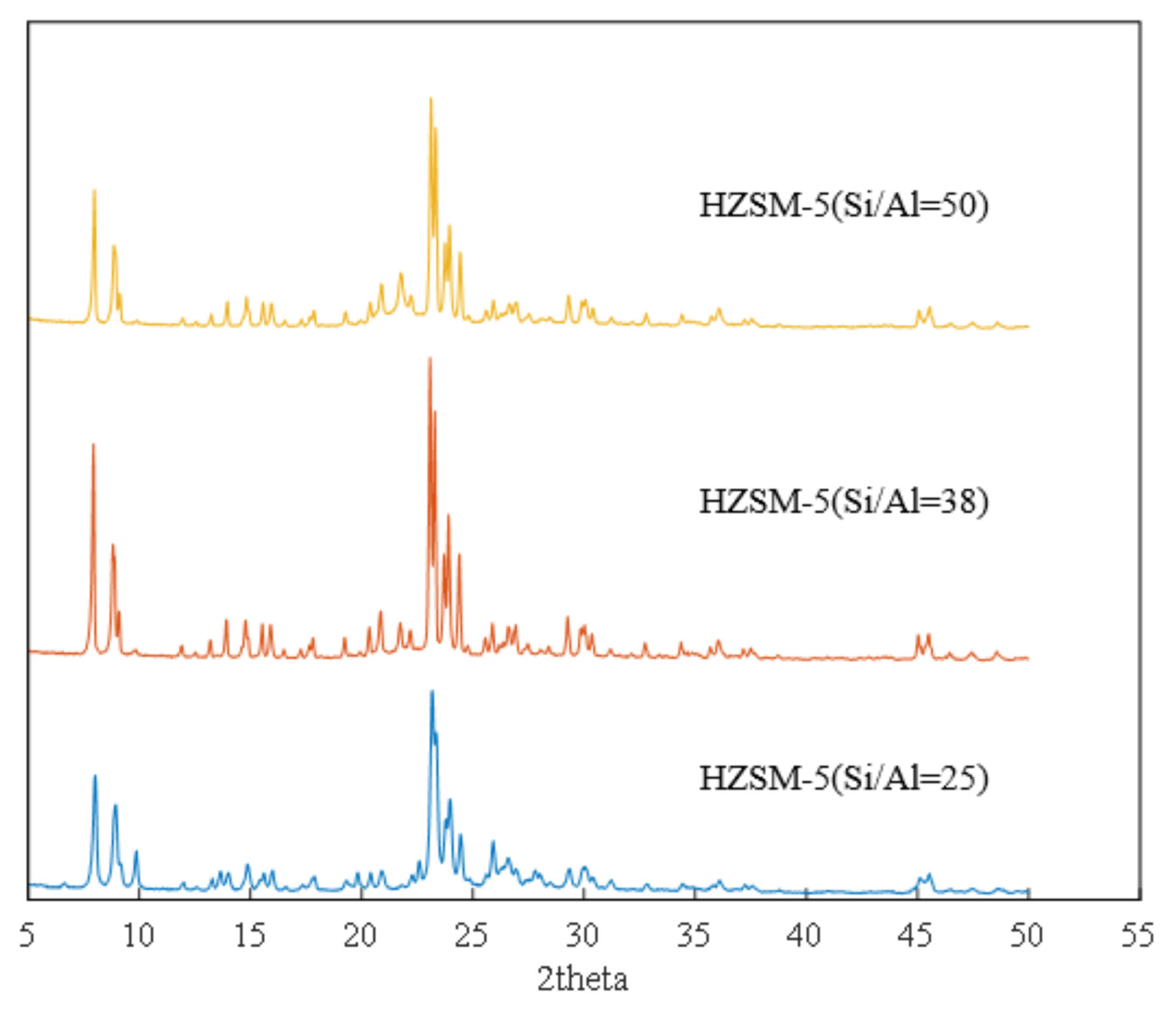

Supplement: Figure 6 — XRD characterization result of HZSM-5 catalyst. [file turkjchem-46-2-446s6.tif]

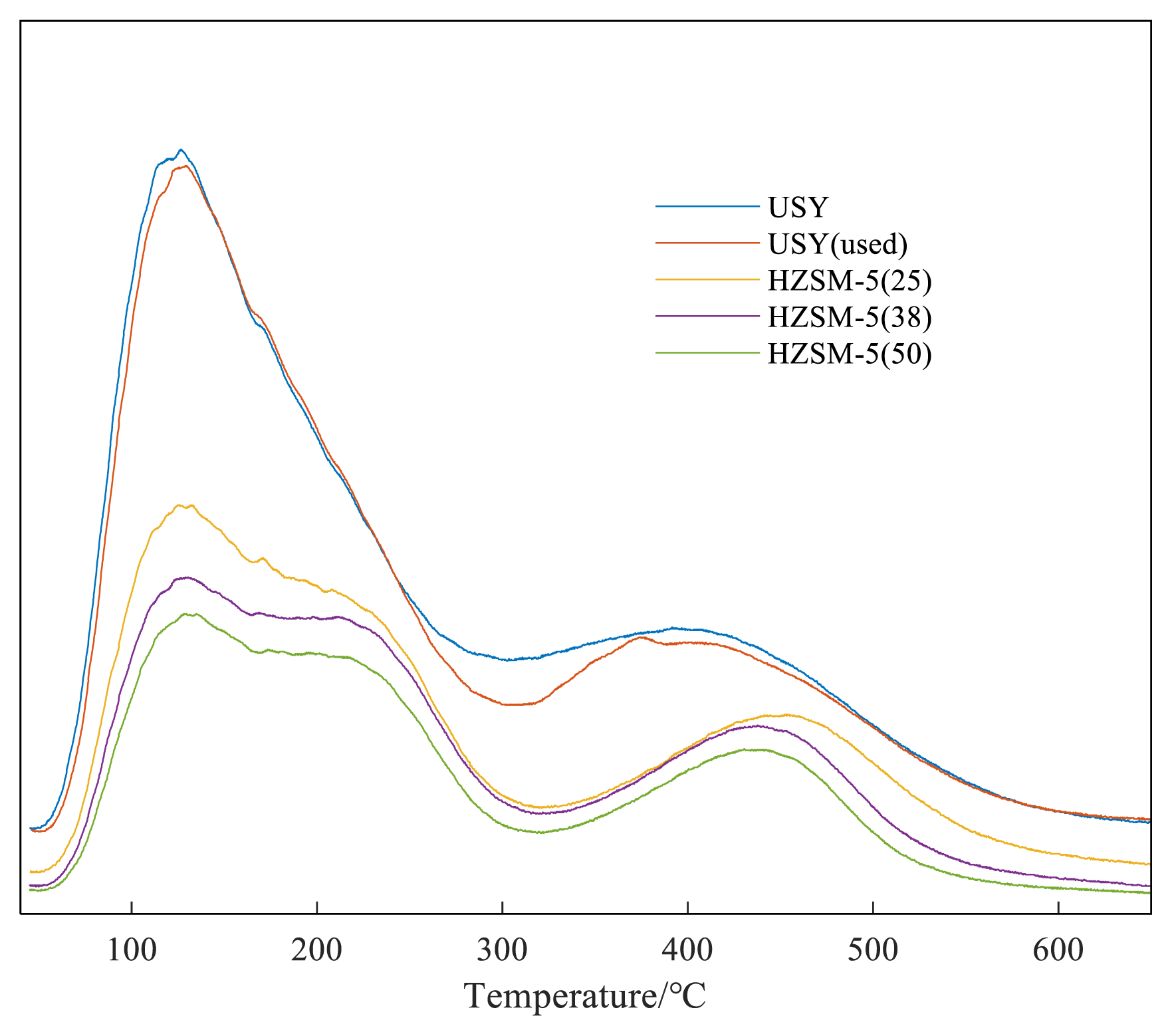

Supplement: Figure 7 — NH3-TPD characterization result of USY catalyst and HZSM-5 catalyst [file turkjchem-46-2-446s7.tif]

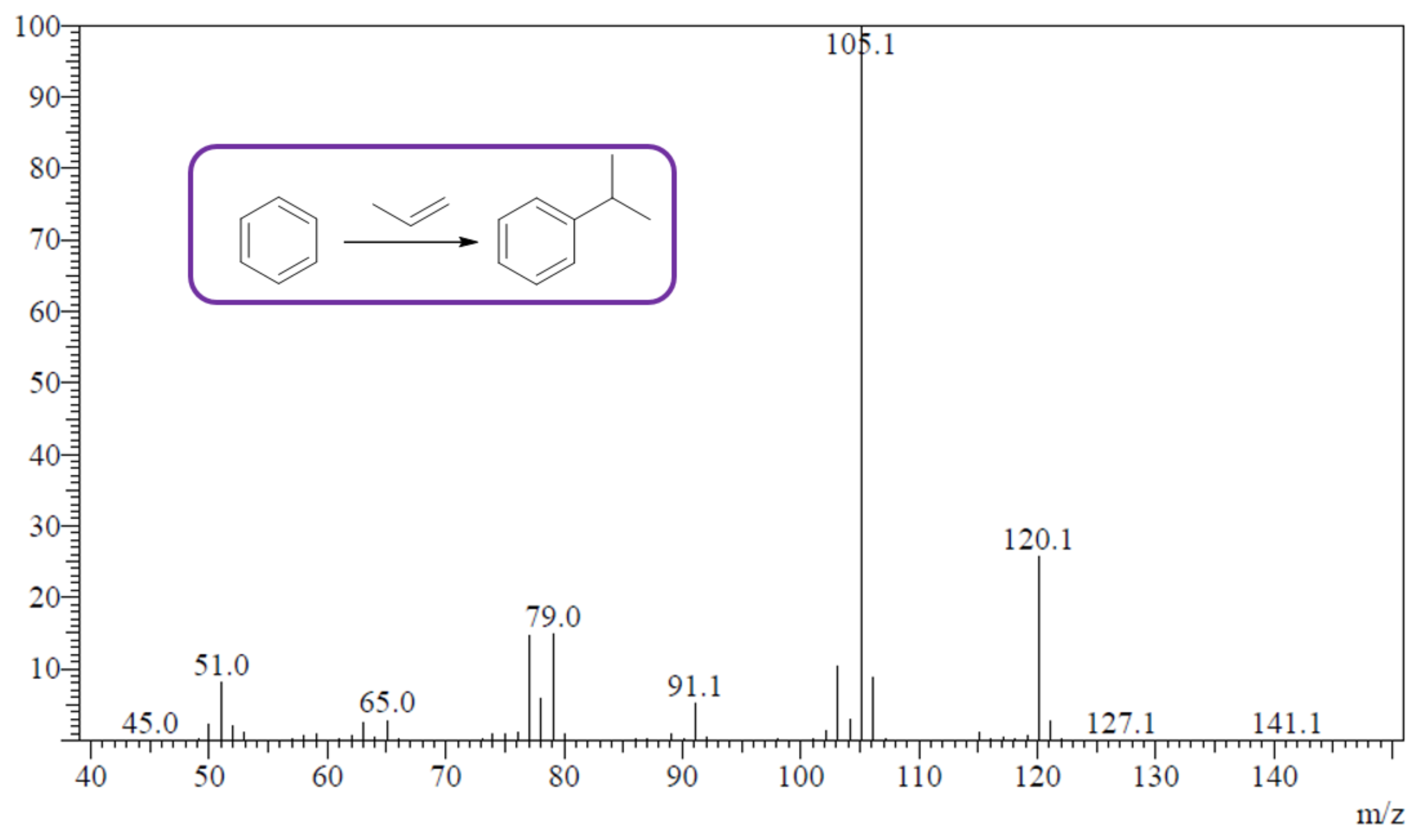

Supplement: Figure 8 — The GC-MS trace of cumene. [file turkjchem-46-2-446s8.tif]

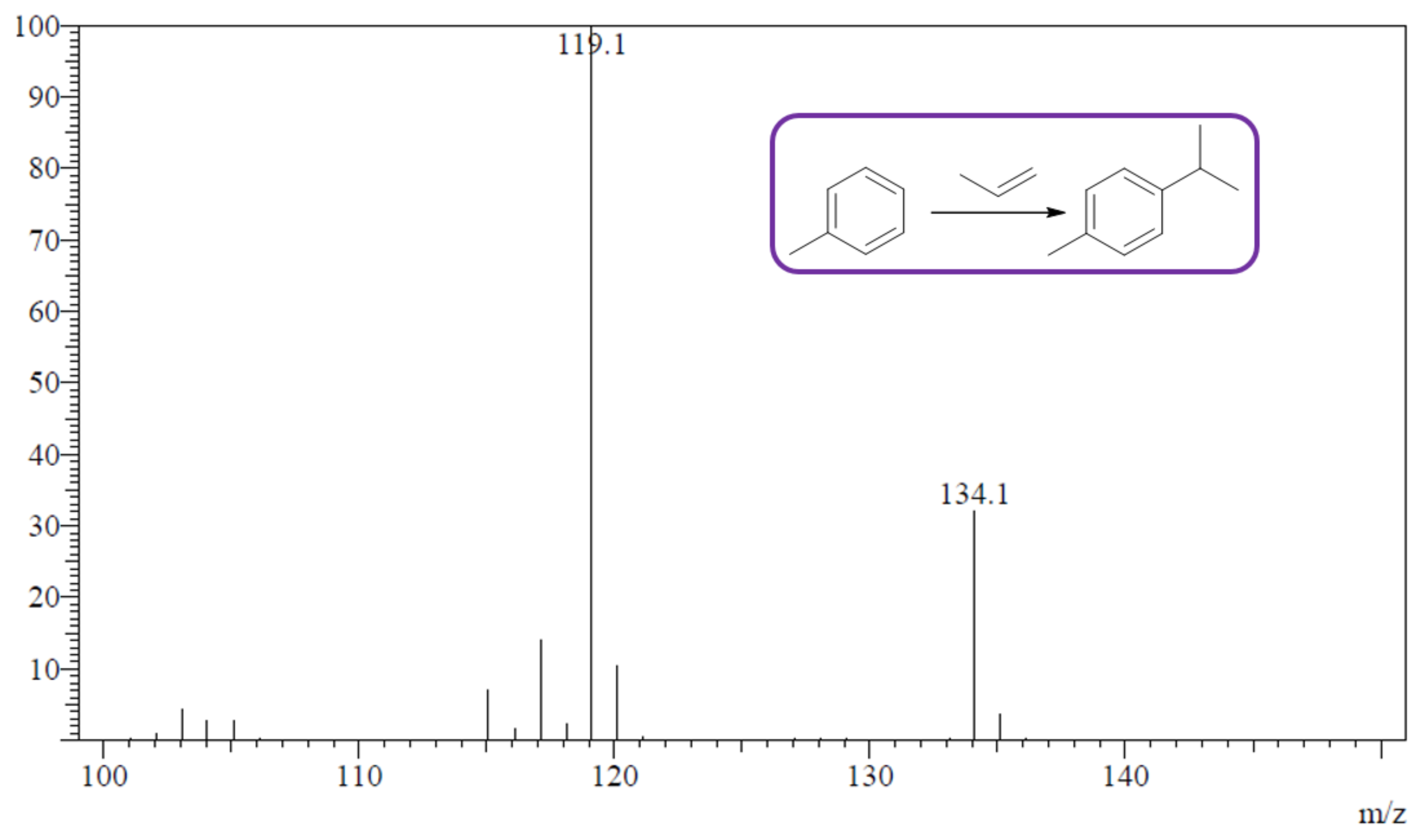

Supplement: Figure 9 — The GC-MS trace of P-cymene. [file turkjchem-46-2-446s9.tif]

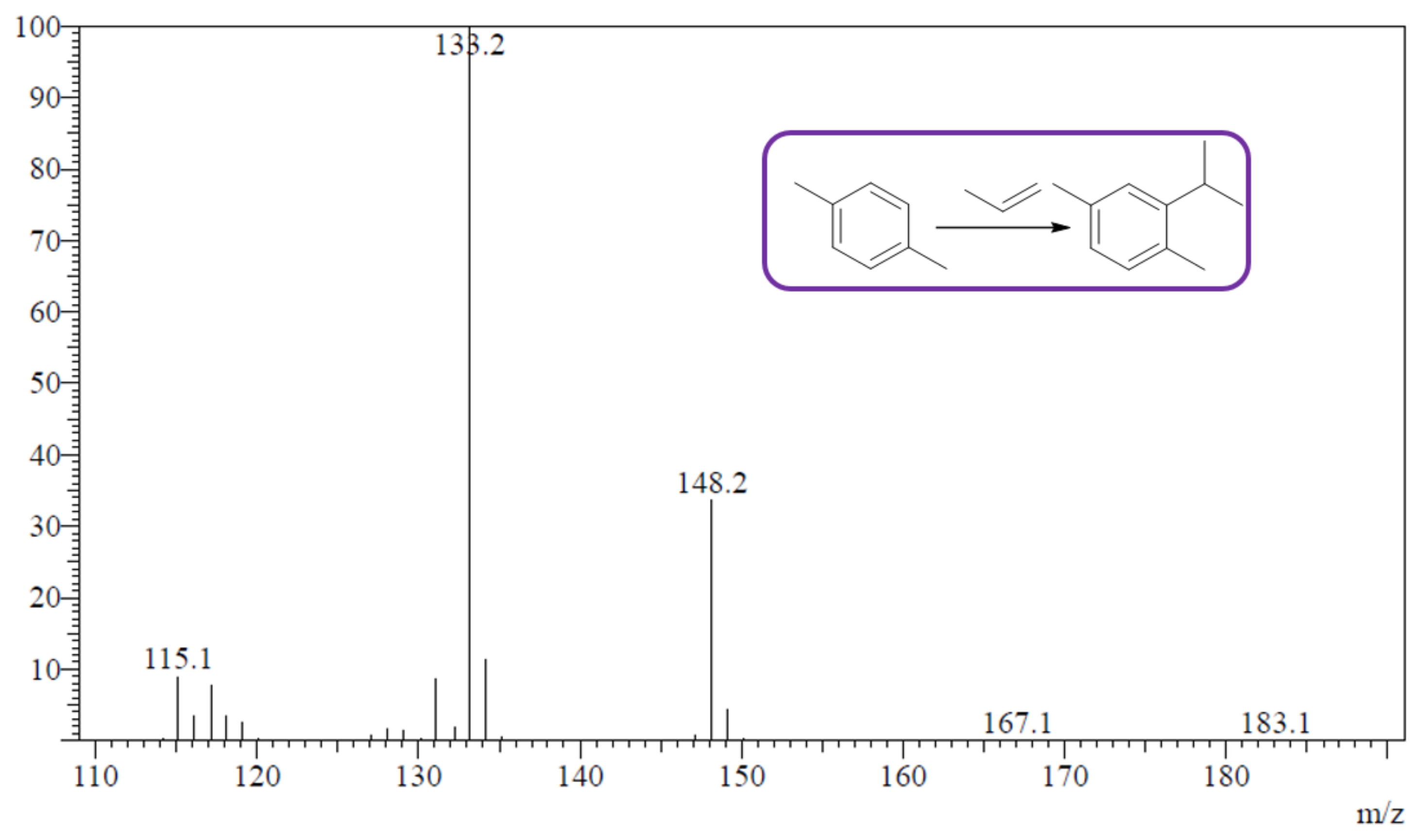

Supplement: Figure 10 — The GC-MS trace of 1-Isopropyl-2,5-dimethylbenzene. [file turkjchem-46-2-446s10.tif]

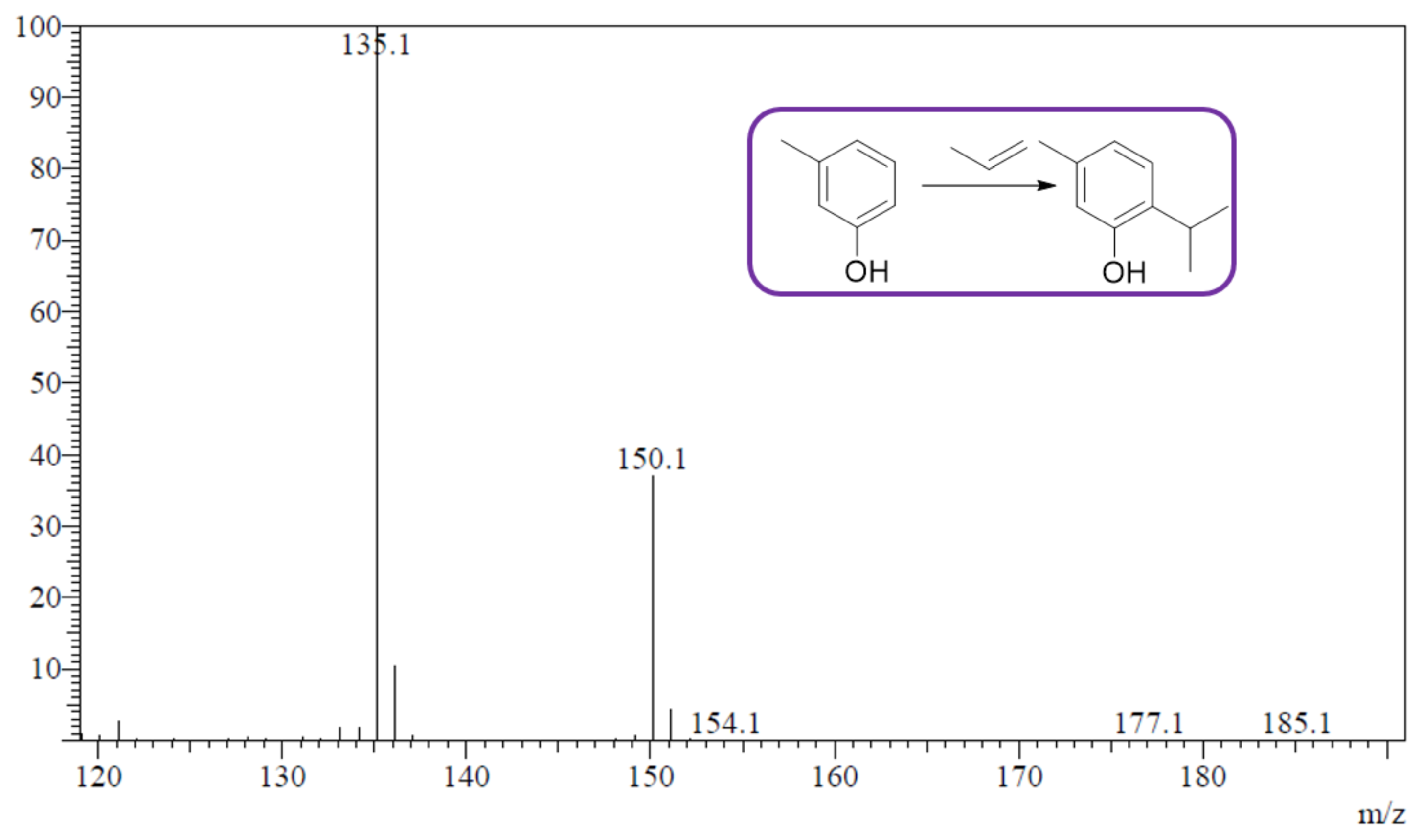

Supplement: Figure 11 — The GC-MS trace of 5-Methyl-2-isopropylphenol. [file turkjchem-46-2-446s11.tif]

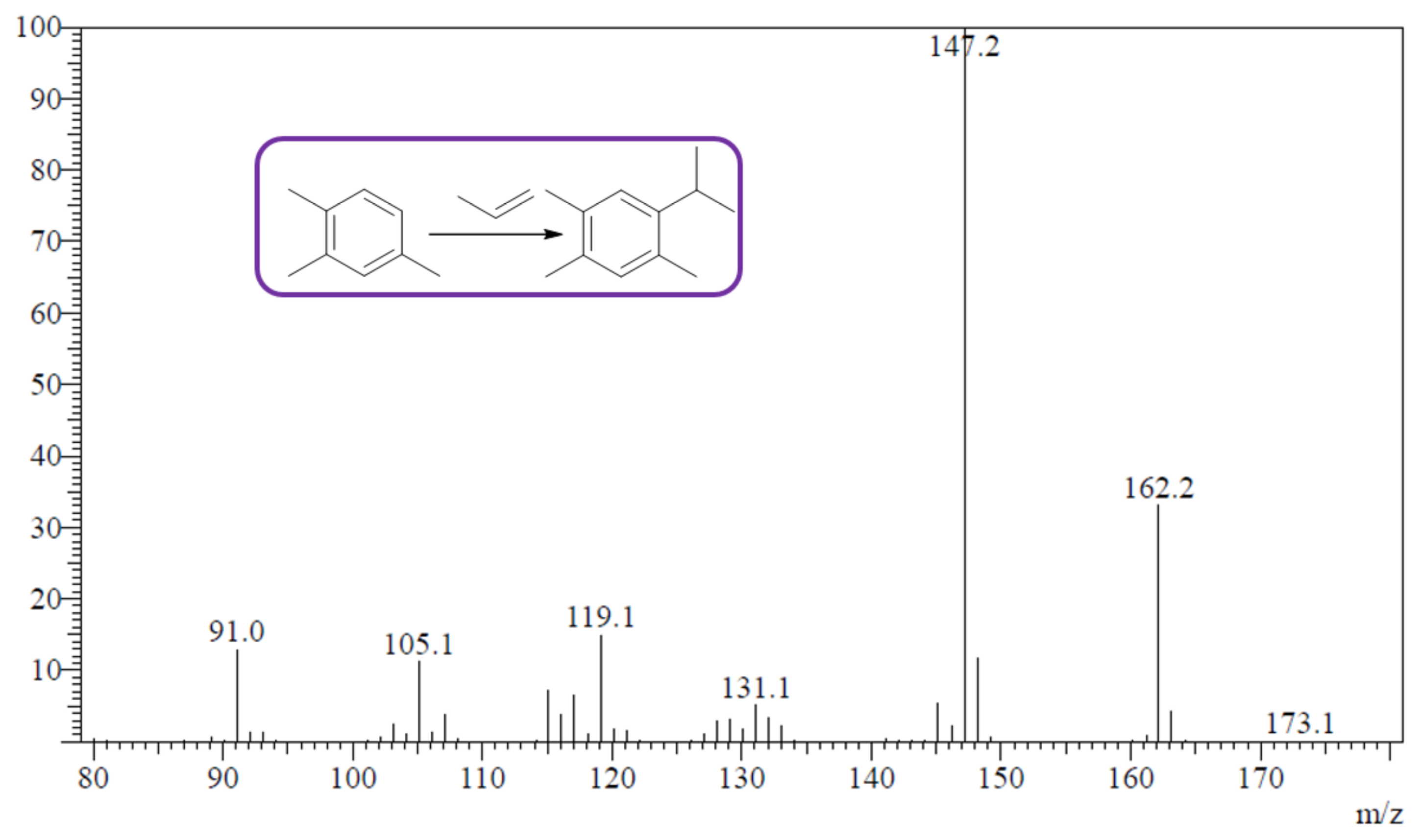

Supplement: Figure 12 — The GC-MS trace of 5-Isopropyltrimethylbenzene. [file turkjchem-46-2-446s12.tif]

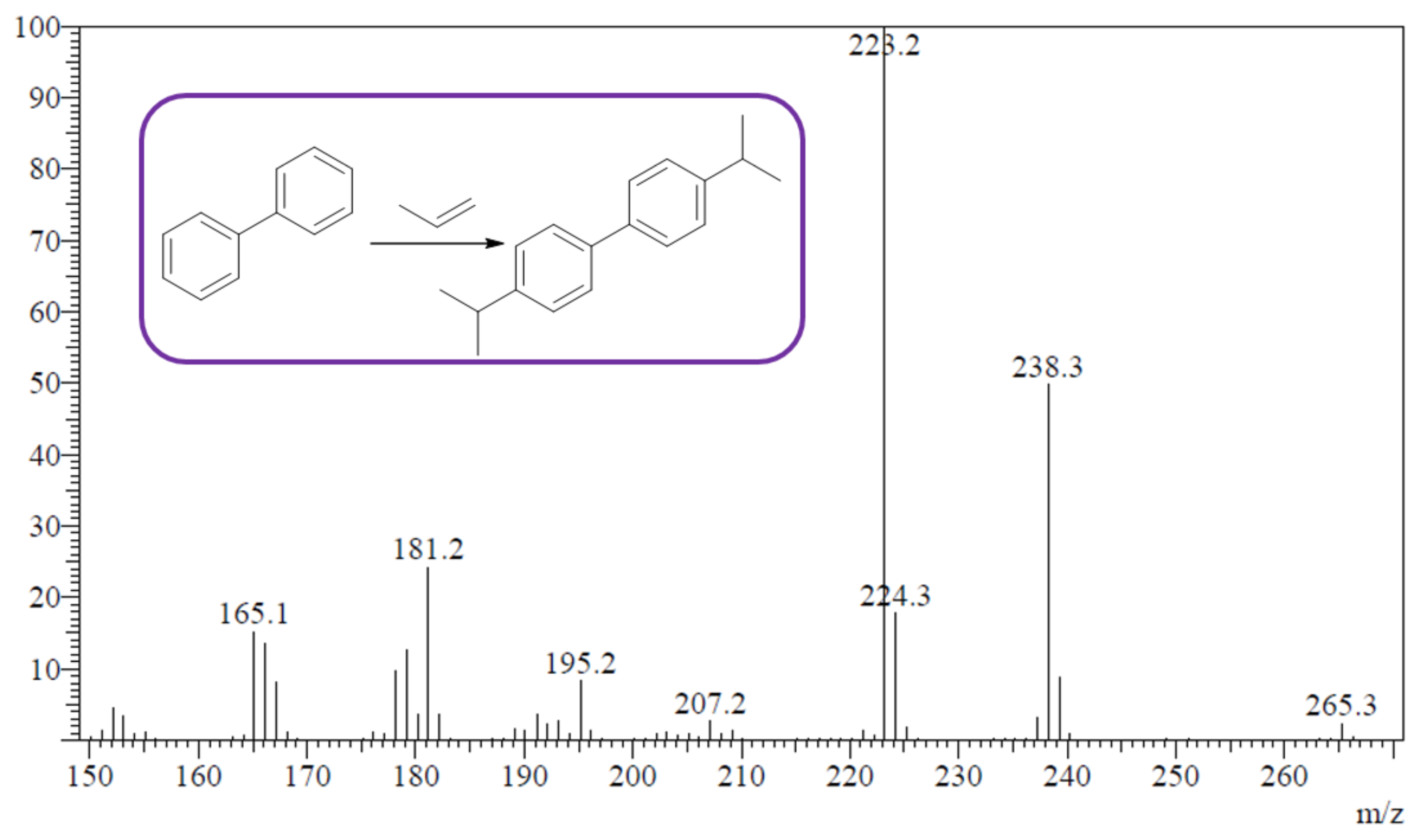

Supplement: Figure 13 — The GC-MS trace of 4,4′-Diisopropenylbiphenyl. [file turkjchem-46-2-446s13.tif]

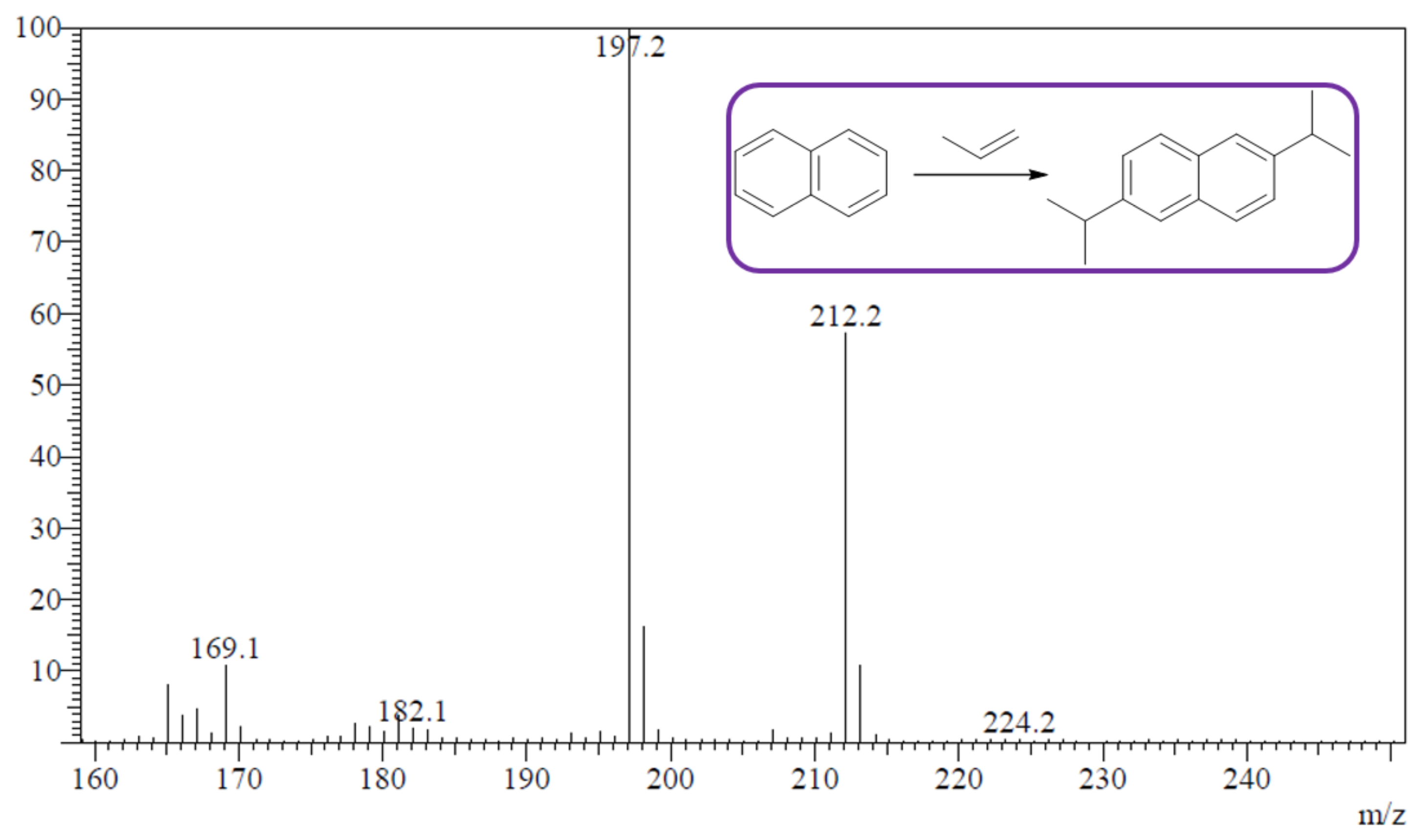

Supplement: Figure 14 — The GC-MS trace of 2,6-Diisopropylnaphthalene. [file turkjchem-46-2-446s14.tif]

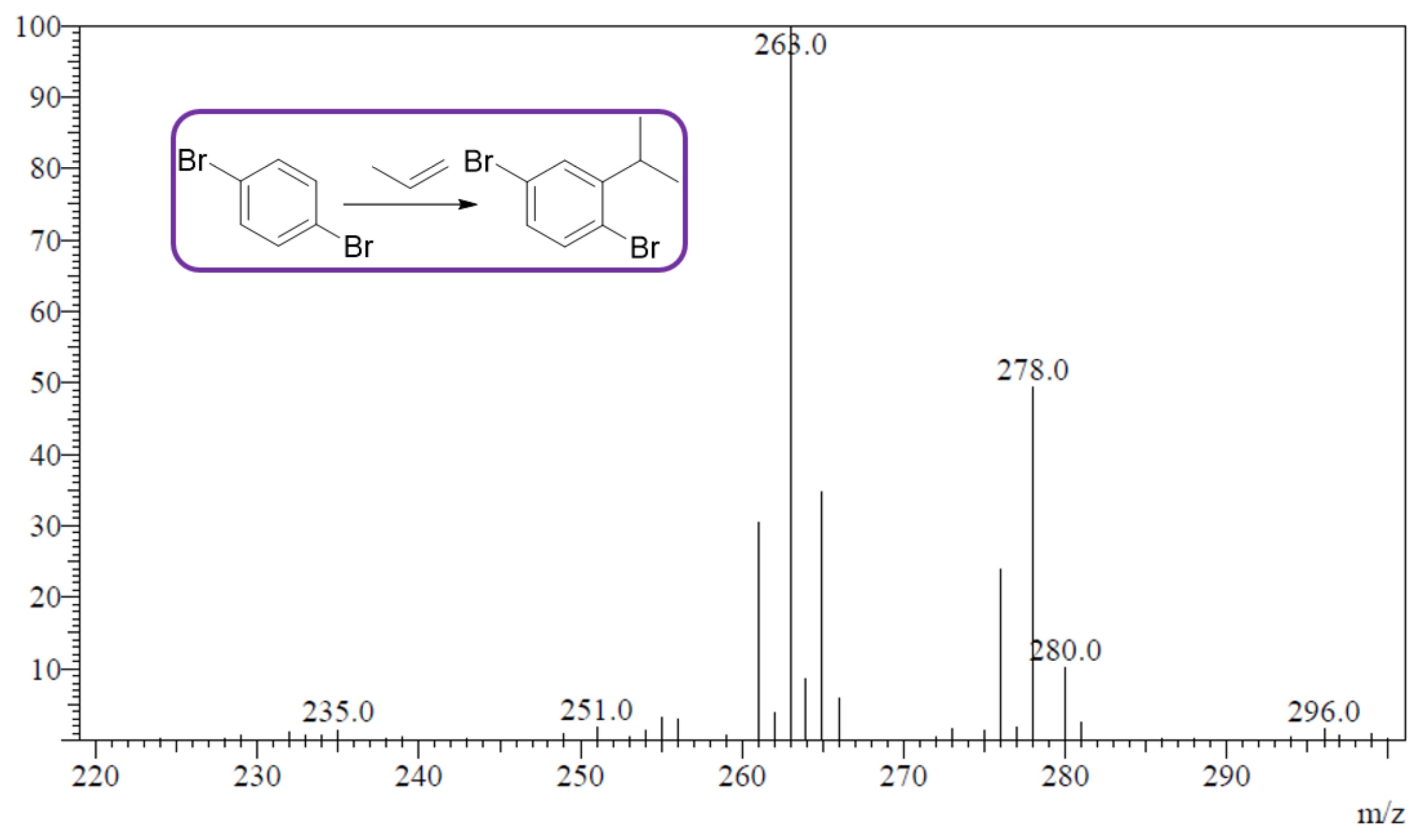

Supplement: Figure 15 — The GC-MS trace of 2,5-Dibromoisopropylbenzene. [file turkjchem-46-2-446s15.tif]

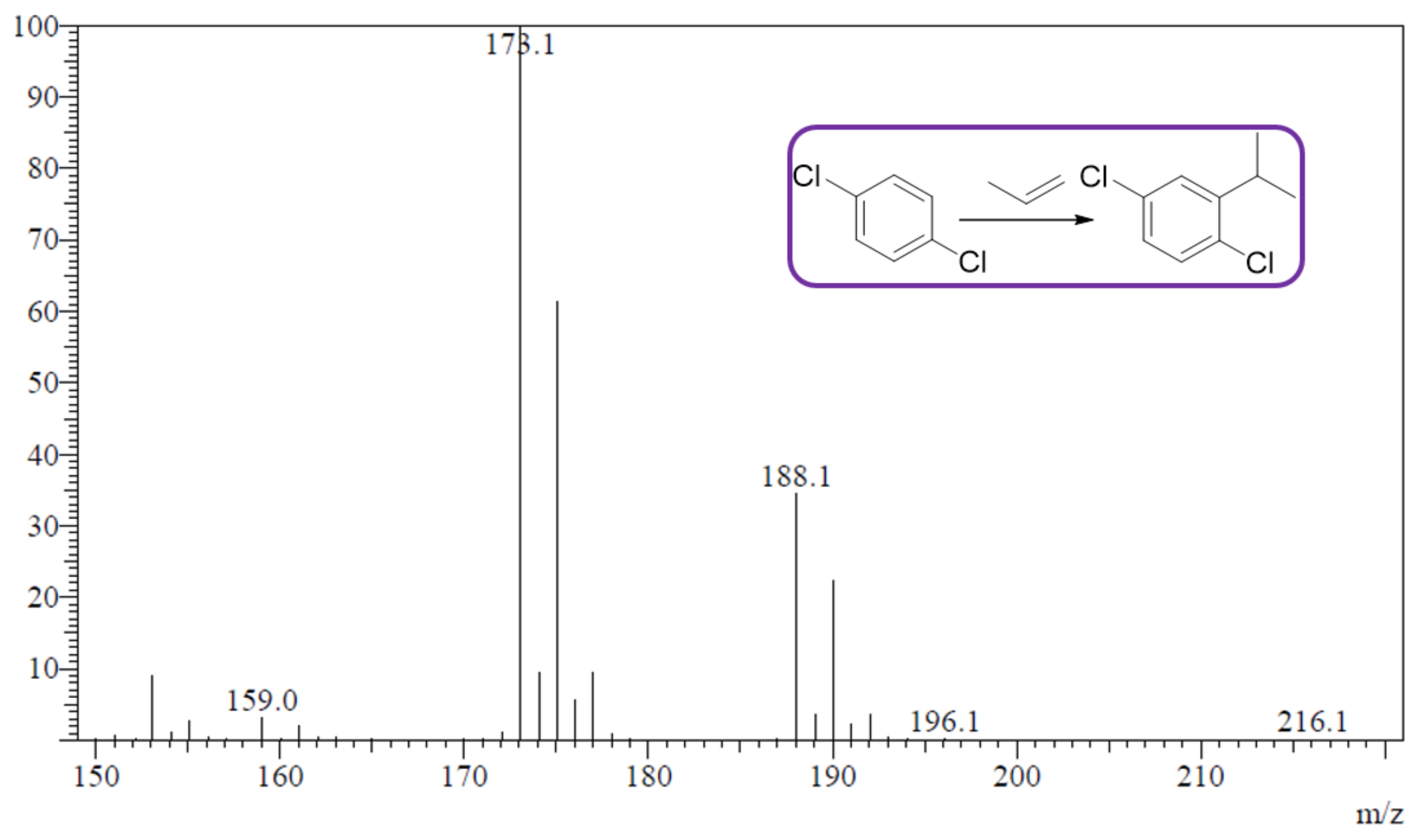

Supplement: Figure 16 — The GC-MS trace of 1,4-Dichloroisopropylbenzene. [file turkjchem-46-2-446s16.tif]

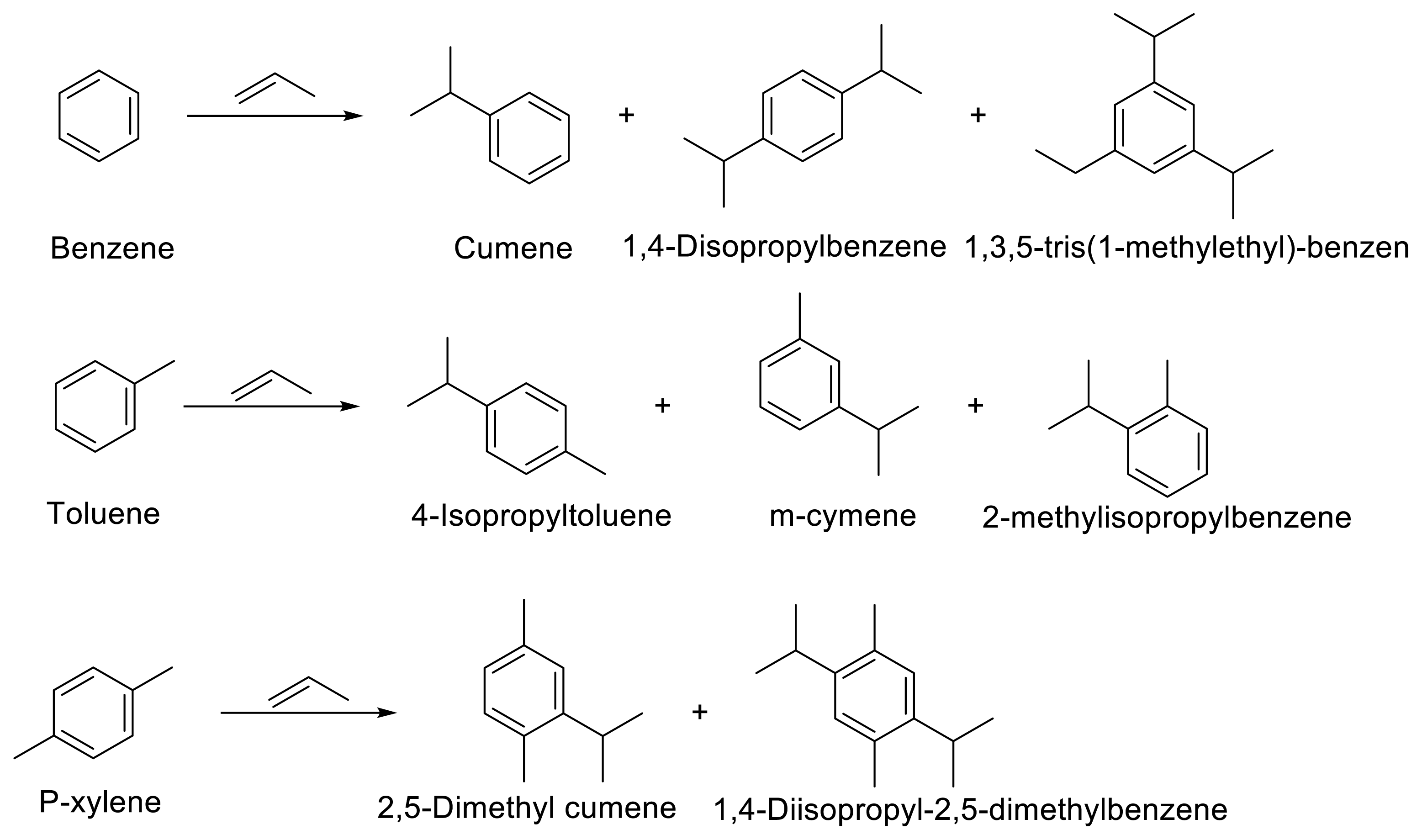

Supplement: Figure 17 — The main products and by-products of the alkylation reaction of benzene, toluene, p-xylene, and propylene. [file turkjchem-46-2-446s17.tif]
